# Modeling the effect of lockdown and social distancing on the spread of COVID-19 in Saudi Arabia

**DOI:** 10.1371/journal.pone.0265779

**Published:** 2022-04-14

**Authors:** Sara K. Al-Harbi, Salma M. Al-Tuwairqi

**Affiliations:** Mathematics Department, King Abdulaziz University, Jeddah, Saudi Arabia; East China Normal University, CHINA

## Abstract

The COVID-19 pandemic spread rapidly worldwide. On September 15, 2021, a total of 546,251 confirmed cases were recorded in Saudi Arabia alone. Saudi Arabia imposed various levels of lockdown and forced the community to implement social distancing. In this paper, we formulate a mathematical model to study the impact of these measures on COVID-19 spread. The model is analyzed qualitatively, producing two equilibrium points. The existence and stability of the COVID-19 free equilibrium and the endemic equilibrium depend on the control reproduction number, $R_{c}$. These results are in good agreement with the numerical experiments. Moreover, the model is fitted with actual data from the COVID-19 dashboard of the Saudi Ministry of Health. We divide the timeline from March 12, 2020, to September 23, 2020, into seven phases according to the varied applications of lockdown and social distancing. We then explore several scenarios to investigate the optimal application of these measures and address whether it is possible to rely solely on social distancing without imposing a lockdown.

## 1 Introduction

At the end of 2019, an infectious disease appeared caused by a new virus called severe acute respiratory syndrome coronavirus 2 (SARS-CoV-2) and was named coronavirus disease 2019 (COVID-19) [1]. The first infection by this virus arose in Wuhan, China, in December 2019 [2]. Then it spread rapidly worldwide; as a result, on March 11, 2020, the World Health Organization (WHO) declared it a pandemic [3]. On September 15, 2021, WHO recorded a total of 225, 680, 357 confirmed cases in the world and 4, 644, 740 deaths [4]. Also, on the same day, the Saudi Ministry of Health (MOH) recorded a total of 546, 251 confirmed cases with a total of 535, 260 recovered and a total of 8, 640 deaths.

World governments have made significant efforts to limit the spread of COVID-19 and reduce the effects of its spread on health care and economics. One of these efforts is implementing preventive measures, such as imposing lockdown, isolation for the infected, social distancing, wearing face masks, and others. In Saudi Arabia, the government imposed preventive measures before recording any infection by COVID-19, such as suspending Umrah from outside the Kingdom on February 27, 2020 [5]. The first confirmed case by COVID-19 was reported in Saudi Arabia on March 2, 2020 [6]. Consequently, the government closed its borders, schools, universities, and workplaces. Also, they suspended all events, activities, and prayers in mosques. Then they applied different levels of lockdown. The first partial lockdown implemented in Saudi Arabia began on March 23, 2020 [7].

Mathematical models are one of the essential tools for analyzing and discussing infectious diseases. It aids in studying the dynamic behavior of the disease [8], predicting the number of cases [9, 10], and determining disease transmission by finding the basic reproduction number. Also, it assists in deciding to impose the best preventive measures for the situation [11]. Many researchers have discussed COVID-19 mathematically with different models. Alzahrani et al. [9] presented four prediction models to predict the daily numbers of COVID-19 infections from April 21 to May 21, 2020, in Saudi Arabia. The authors conducted this study before the Hajj season, where the Kingdom was supposed to receive large numbers of Muslims from different countries of the world in mid-July to perform the Hajj. The number of pilgrims reached 2,489,406 in 2019, making the Kingdom more susceptible to infection [12]. The results predicted in the study showed an increase in the number of cases in the Kingdom, and it may reach more than 7668 new cases per day if strict preventive measures are not imposed. Also, Alboaneen et al. [10] provided two models to predict the number of cases in Saudi Arabia, which are the Logistic Growth and the Susceptible-Infected-Recovered model.

The effectiveness and impact of social distancing in limiting the spread of COVID-19 were analyzed by researchers. For instance, Aldila et al. in [13] formulated a compartment model for studying the impact of social distancing and rapid COVID-19 testing in Jakarta. The proposed model was fitted to cumulative data from March 3 to May 10, 2020. They discussed the plan created by the Jakarta government to relax the strict social distancing, which consists of five phases. By assuming that the transmission rate is a step function, the increase in relaxing the social distancing is offset by the increase in the transmission rate. They concluded that strict social distancing is an essential measure that reduces infections and delays the time of disease outbreak. Also, Saif Ullah and Khan [14] agreed with this result by studying a model that includes social distancing, isolation, and quarantine with the case study of Pakistan. The authors considered the effective contact rate as a measure of social distancing. Then, they analyzed mild, moderate, and strict social distancing cases by reducing the effective contact rate by 10%, 30%, and 35%, respectively.

On the other hand, some studies expressed the social distancing as parameters in the mathematical model [15–18]. Researchers in [15–17] determined the value of social distancing from fitting the proposed model to actual data, while Kennedy et al. [18] determined it through mobility data trends from Apple Maps. Moreover, Peter et al. [16] imposed two parameters, one for physical distancing, which is at least two meters, and the other for effective use of a face mask and hand sanitizer.

When COVID-19 spread significantly, many countries imposed the lockdown as a preventive measure to limit the spread of the virus. Accordingly, researchers discussed the lockdown in their models. Ibarra-Vega in [19] built a mathematical model for the effectiveness of lockdown and used piecewise functions to discuss three scenarios for lockdown. Ibarra-Vega concluded that all scenarios are good and help reduce the number of infections and deaths. In addition, it is necessary to discuss the effectiveness of the lockdown in different countries with scenarios that fit the different characteristics of each country, such as the population density, the economy, and health systems, since the characteristics determine the level of communication between people of society. For example, Alrashed et al. in [20] discussed different levels of lockdown in Saudi Arabia. They added a parameter that expresses the lockdown to the Susceptible-Exposed-Infected-Recovered model. The study concluded that the increase in lockdown implementation decreases cases. However, in the absence of lockdown, they expected the number of cases to reach 2 million during the peak of the spread, which is about two months from March 25, 2020. Also, Fanelli et al. [21] formulated a mathematical model and reported their prediction of the number of cases in China, Italy, and France. They expressed the infection rate as a variable with time to study the effect of imposing the lockdown in Italy and predict the number of infected and dying. After imposing the lockdown in Italy on March 8, 2020, the value of the infection rate varied from its imposed value, affected by the level of the applied procedures.

At the beginning of the emergence of COVID-19, Saudi Arabia imposed different levels of lockdown and obligated members of the society to apply social distancing. Our goal in this paper is to study the impact of lockdown and social distancing on the spread of COVID-19 in Saudi Arabia. This study discusses the optimal measures and addresses whether it is possible to rely on social distancing only without imposing a lockdown. This work is organized as follows. In section 2, we formulate the model and show that the model is well-posed. Then, in section 3, we present the qualitative analysis of the model, the existence of equilibrium points, and their local and global stability. In addition, in section 4, we present the numerical analysis of the model, which includes the model fitting to cumulative data of COVID-19 cases by the Saudi Ministry of Health (MOH) and estimation of parameters. Moreover, we confirm our qualitative results by performing numerical experiments and presenting the sensitivity of the control reproduction number. In addition, we analyze different scenarios for lockdown and social distancing.

## 2 Mathematical model

The model divides the total size of the Saudi population, *N*, into four compartments: susceptible, exposed, infected, and recovered. *S* is the class of individuals susceptible to COVID-19 disease. *E* is the exposed class, individuals who caught the virus but with no symptoms, that is, undergoing an incubation period. *I* is the class of COVID-19 infected individuals. *R* is the recovered class, individuals who recovered and are immune. The state variables *S*, *E*, *I*, and *R* are functions of the independent variable time *t*. Individuals move from the susceptible class to the exposed compartment after interacting with infected individuals at transmission rate *β*. The transmission rate may be controlled by implying direct measures such as lockdown and social distancing. In our model, we combine the two measures to study and compare their effectiveness in halting the spread of COVID-19. The lockdown is represented by the function *ρ*(*t*) ∈ (0, 1]. If *ρ* = 1, then there is no lockdown; conversely, full lockdown is when *ρ* ≈ 0. Meanwhile, the social distancing is expressed in the model by the function *SD*(*t*) ∈ [0, 1). A complete social distancing is carried out when *SD* ≈ 1. We assume that social distancing means staying at home, keeping a safe space when communicating with other individuals, and wearing a face mask. At the end of the incubation period, 1/*γ*, individuals move from the exposed compartment to the infected compartment. Unfortunate infected individuals die due to COVID-19 at a rate of *d*. Others may recover and leave the infected compartment after a period of 1/*δ*. Finally, we assume the natural death rate from all compartments is *μ*, and *η* is the natural birth rate.

**Fig 1:**
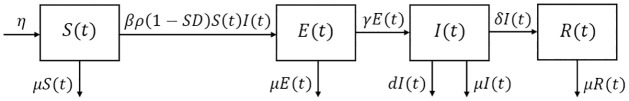
Flowchart of the model.

The dynamics of the model are illustrated in Fig 1 and expressed mathematically by the following nonlinear system of ODEs:

$$\begin{array}{l} \begin{array}{l} \frac{dS}{dt}=\eta -\beta \rho (1-SD)SI-\mu S,\\ \frac{dE}{dt}=\beta \rho (1-SD)SI-(\gamma +\mu )E,\\ \frac{dI}{dt}=\gamma E-(\delta +d+\mu )I,\\ \frac{dR}{dt}=\delta I-\mu R, \end{array} \end{array}$$ (1)

where all parameters are positive and *N* = *S*(*t*) + *E*(*t*) + *I*(*t*) + *R*(*t*).

In the beginning, we shall prove that system (1) is well-posed, and the state variables are epidemiologically meaningful, i.e., non-negative and bounded.

**Theorem 1**. *If* $\left(S,E,I,R\right)\in R_{\geq 0}^{4}$ , *then the set*

$$\begin{array}{c} \Omega =\{\left(S,E,I,R\right)\in R_{\geq 0}^{4}:0\leq N\leq \frac{\eta }{\mu }\} \end{array}$$

*is positively invariant for model* (1).

**Proof.** Let (*S*(0), *E*(0), *I*(0), *R*(0)) ∈ Ω. We have

$$\begin{array}{cc} \frac{dS}{dt}{|}_{S=0} & =\eta >0,\\ \frac{dE}{dt}{|}_{E=0} & =\beta \rho (1-SD)SI\geq 0,\;\text{for}\;\text{all}\;S,I\geq 0,\\ \frac{dI}{dt}{|}_{I=0} & =\gamma E\geq 0,\;\text{for}\;\text{all}\;E\geq 0,\\ \frac{dR}{dt}{|}_{R=0} & =\delta I\geq 0,\;\text{for}\;\text{all}\;I\geq 0. \end{array}$$

Thus, for *t* ≥ 0 all non-negative solutions remain non-negative. Next, we combine all equations of model (1), we get

$$\begin{array}{c} \frac{dN}{dt}=\eta -dI-\mu N\leq \eta -\mu N. \end{array}$$

Using the integrating factor method [22], we multiply the above inequality by the integrating factor (*e*^*μr*^),

$$\begin{array}{c} \frac{d}{dr}\left[e^{\mu r}N\left(r\right)\right]\leq \eta e^{\mu r}. \end{array}$$

Integrating both sides over the time interval [0, *t*], we obtain

$$\begin{array}{c} N\left(t\right)\leq \frac{\eta }{\mu }+\left[N\left(0\right)-\frac{\eta }{\mu }\right]e^{-\mu t}. \end{array}$$

Thus,

$$\begin{array}{c} \lim_{t\to \infty }Sup\left[N\left(t\right)\right]\leq \frac{\eta }{\mu }. \end{array}$$ (2)

Hence, all solutions of model (1) are bounded and non-negative for all *t* ≥ 0. Therefore, Ω is positively invariant.

## 3 Qualitative analysis

In this section, we discuss model (1) qualitatively. We determine the equilibrium points and the expression of the basic reproduction number. In addition, we investigate the local and global stability of the equilibrium points.

### 3.1 Equilibrium points and basic reproduction number

To determine the equilibrium points of model (1), we set the derivatives equal to zero. We get,

$$\begin{array}{cc} \eta -\beta \rho (1-SD)SI-\mu S & =0,\\ \beta \rho (1-SD)SI-(\gamma +\mu )E & =0,\\ \gamma E-(\delta +d+\mu )I & =0,\\ \delta I-\mu R & =0. \end{array}$$ (3)

The analytic solution of system (3) yields two equilibrium points: the COVID-19 free equilibrium point, *P*_0_ = (*η*/*μ*, 0, 0, 0), which is always present, and the COVID-19 endemic equilibrium point, *P*_1_ = (*S*_1_, *E*_1_, *I*_1_, *R*_1_), where

$$\begin{array}{cccc} S_{1} & =\frac{\eta }{\mu R_{c}}, & I_{1} & =\frac{\mu (R_{c}-1)}{\beta \rho (1-SD)},\\ E_{1} & =\frac{\mu \left(\delta +d+\mu \right)\left(R_{c}-1\right)}{\gamma \beta \rho (1-SD)}, & R_{1} & =\frac{\delta (R_{c}-1)}{\beta \rho (1-SD)}. \end{array}$$

Here $R_{c}=\left[\beta \rho \left(1-SD\right)\eta \gamma \right]/\left[\mu \left(\gamma +\mu \right)\left(\delta +d+\mu \right)\right]$. The endemic equilibrium *P*_1_ exists only if $R_{c}>1$.

#### Basic reproduction number

The basic reproduction number ($R_{0}$) is the threshold condition for an epidemic to occur. In our model, $R_{0}$ represents the secondary cases of COVID-19 produced from one infectious individual in a population containing only susceptible individuals. To find $R_{0}$, we use the next generation matrix method [23]. Let $O={\left(E,I\right)}^{T}$, then the second and third equations in (1) can be rewritten as $\dot{O}=F\left(O\right)-V\left(O\right)$, where

$$\begin{array}{c} F=[\begin{array}{c} \beta \rho (1-SD)SI\\ 0 \end{array}], \end{array}$$

and

$$\begin{array}{c} V=[\begin{array}{c} (\gamma +\mu )E\\ -\gamma E+(\delta +d+\mu )I \end{array}]. \end{array}$$

We compute the Jacobian matrix of F and V at the COVID-19 free equilibrium point *P*_0_, we get, respectively,

$$\begin{array}{c} F=[\begin{array}{cc} 0 & \frac{\beta \rho (1-SD)\eta }{\mu }\\ 0 & 0 \end{array}], \end{array}$$

and

$$\begin{array}{c} V=[\begin{array}{cc} \gamma +\mu & 0\\ -\gamma & \delta +d+\mu \end{array}]. \end{array}$$

The next generation matrix is

$$\begin{array}{c} FV^{-1}=[\begin{array}{cc} \frac{\beta \rho (1-SD)\eta \gamma }{\mu (\gamma +\mu )(\delta +d+\mu )} & \frac{\beta \rho (1-SD)\eta }{\mu (\delta +d+\mu )}\\ 0 & 0 \end{array}]. \end{array}$$

Hence, the basic reproduction number obtained from the next generation matrix, is the spectral radius of matrix *FV*^−1^, that is,

$$\begin{array}{c} R_{c}=\frac{\beta \rho (1-SD)\eta \gamma }{\mu (\gamma +\mu )(\delta +d+\mu )}. \end{array}$$

Here we call this expression by the control reproduction number because our model includes two control measures, the lockdown *ρ* and the social distancing (*SD*), where $R_{c}=\rho \left(1-SD\right)R_{0}$. The terms in $R_{0}$ are explained further as follows. The term that expresses the incidence of new infections by infected individuals is *βSI*. Thus, the number of secondary cases by one infectious individual (*I* = 1) in a population containing only susceptible individuals is *βS*_0_, where *S*_0_ = *η*/*μ*. Moreover, 1/(*δ* + *d* + *μ*) represents the average time spent by one infectious individual in the infected compartment. Also, *γ*/(*γ* + *μ*) is the proportion of newly infected individuals that survived the incubation period.

### 3.2 Local stability analysis

We investigate the local stability of the equilibrium points of model (1) by using the linearization method [24].

**Theorem 2**. *The COVID-19 free equilibrium point P*_0_ *is locally asymptotically stable if* $R_{c}<1$.

**Proof**. The Jacobian matrix of model (1) at *P*_0_ is given by

$$\begin{array}{c} J\left(P_{0}\right)=[\begin{array}{cccc} -\mu & 0 & \frac{-\beta \rho (1-SD)\eta }{\mu } & 0\\ 0 & -(\gamma +\mu ) & \frac{\beta \rho (1-SD)\eta }{\mu } & 0\\ 0 & \gamma & -(\delta +d+\mu ) & 0\\ 0 & 0 & \delta & -\mu \end{array}]. \end{array}$$

Solving the characteristic equation ∣*J*(*P*_0_) − λ*I*∣ = 0, we get the eigenvalues: λ_1,2_ = −*μ*, and λ_3,4_ satisfy the following equation

$$\begin{array}{c} \lambda^{2}+a_{1}\lambda +a_{2}=0, \end{array}$$

where

$$\begin{array}{cc} a_{1} & =\gamma +\delta +d+2\mu ,\\ a_{2} & =\left(\gamma +\mu \right)\left(\delta +d+\mu \right)-\frac{\beta \rho (1-SD)\eta \gamma }{\mu }. \end{array}$$

It is clear that λ_1,2_ are negative. Using Routh-Hurwitz criteria [25], λ_3,4_ are negative if *a*_1_ > 0, and *a*_2_ > 0. Clearly, *a*_1_ > 0 and to get *a*_2_ > 0 we must have (*γ* + *μ*)(*δ* + *d* + *μ*) > *βρ*(1 − *SD*)*ηγ*/*μ*, that is, $R_{c}<1$. Hence, *P*_0_ is locally asymptotically stable if $R_{c}<1$.

**Theorem 3**. *The COVID-19 endemic equilibrium point P*_1_ *is locally asymptotically stable if* $R_{c}>1$ .

**Proof**. The Jacobian matrix of model (1) at *P*_1_ is given by

$$\begin{array}{c} J\left(P_{1}\right)=[\begin{array}{cccc} -\beta \rho \left(1-SD\right)I_{1}-\mu & 0 & -\beta \rho \left(1-SD\right)S_{1} & 0\\ \beta \rho \left(1-SD\right)I_{1} & -(\gamma +\mu ) & \beta \rho \left(1-SD\right)S_{1} & 0\\ 0 & \gamma & -(\delta +d+\mu ) & 0\\ 0 & 0 & \delta & -\mu \end{array}]. \end{array}$$

Solving the characteristic equation ∣*J*(*P*_1_) − λ*I*∣ = 0 yields λ_1_ = −*μ* which is negative, and the remaining eigenvalues λ_2,3,4_ satisfy the following equation

$$\begin{array}{c} \lambda^{3}+a_{1}\lambda^{2}+a_{2}\lambda +a_{3}=0, \end{array}$$

where

$$\begin{array}{c} \begin{array}{cc} a_{1} & =\beta \rho \left(1-SD\right)I_{1}+\gamma +\delta +d+3\mu \\ & =\mu R_{c}+\gamma +\delta +d+2\mu , \end{array}\\ \begin{array}{cc} a_{2} & =\beta \rho \left(1-SD\right)\left(\gamma +\delta +d+2\mu \right)I_{1}+\mu \left(\gamma +\delta +d+2\mu \right)+\left(\gamma +\mu \right)\left(\delta +d+\mu \right)\\ & \;-\gamma \beta \rho \left(1-SD\right)S_{1}\\ & =\mu \left(\gamma +\delta +d+2\mu \right)R_{c}, \end{array}\\ \begin{array}{cc} a_{3} & =\left(\gamma +\mu \right)\left(\delta +d+\mu \right)\left(\mu +\beta \rho \left(1-SD\right)I_{1}\right)-\gamma \mu \beta \rho \left(1-SD\right)S_{1}\\ & =\mu \left(\gamma +\mu \right)\left(\delta +d+\mu \right)\left(R_{c}-1\right). \end{array} \end{array}$$

We use the Routh-Hurwitz criteria to determine the sign of the remaining eigenvalues λ_2,3,4_. They are negative if *a*_1_ > 0, *a*_3_ > 0, and *a*_1_ *a*_2_ − *a*_3_ > 0. Since *P*_1_ exists if $R_{c}>1$, then *a*_1_ > 0, and *a*_3_ > 0. Also, we have

$$\begin{array}{c} \begin{array}{cc} a_{1}a_{2}-a_{3} & =\mu^{2}\left(\gamma +\delta +d+2\mu \right)R_{c}^{2}+\mu {\left(\gamma +\delta +d+2\mu \right)}^{2}R_{c}\\ & \;-\mu \left(\gamma +\mu \right)\left(\delta +d+\mu \right)\left(R_{c}-1\right)\\ & =\mu^{2}\left(\gamma +\delta +d+2\mu \right)R_{c}^{2}+\mu {\left(\gamma +\mu \right)}^{2}R_{c}+2\mu \left(\gamma +\mu \right)\left(\delta +d+\mu \right)R_{c}\\ & \;+\mu {\left(\delta +d+\mu \right)}^{2}R_{c}-\mu \left(\gamma +\mu \right)\left(\delta +d+\mu \right)R_{c}+\mu \left(\gamma +\mu \right)\left(\delta +d+\mu \right)\\ & =\mu^{2}\left(\gamma +\delta +d+2\mu \right)R_{c}^{2}+\mu {\left(\gamma +\mu \right)}^{2}R_{c}+\mu \left(\gamma +\mu \right)\left(\delta +d+\mu \right)R_{c}\\ & \;+\mu {\left(\delta +d+\mu \right)}^{2}R_{c}+\mu \left(\gamma +\mu \right)\left(\delta +d+\mu \right). \end{array} \end{array}$$

Thus, *a*_1_ *a*_2_ − *a*_3_ > 0. Hence, *P*_1_ is locally asymptotically stable if $R_{c}>1$.

### 3.3 Global stability analysis

We discuss the global stability of the equilibrium points of model (1) using the Lyapunov and Krasovkii–LaSalle stability theorem [26–28]. Moreover, we define a function *W* as

$$\begin{array}{c} W(u)=u-1-\text{ln}u. \end{array}$$

It is obvious that *W*(*u*) > 0 for all *u* > 0 and *W*(1) = 0. This function will be utilized in the proof of the global stability of the equilibrium points.

**Theorem 4**. *The COVID-19 free equilibrium point P*_0_ *is globally asymptotically stable if* $R_{c}<1$ .

**Proof**. Define the Lyapunov function *L*_0_(*S*, *E*, *I*, *R*) as:

$$\begin{array}{c} L_{0}=S_{0}W\left(S/S_{0}\right)+E+\frac{\left(\gamma +\mu \right)}{\gamma }I+\frac{\eta \beta \rho (1-SD)}{\delta \mu }\left(1-R_{c}\right)R. \end{array}$$

Clearly, *L*_0_ is positive definite since *L*_0_(*S*, *E*, *I*, *R*) > 0 for all (*S*, *E*, *I*, *R*) ∈ Ω, and *L*_0_(*P*_0_) = *L*_0_(*S*_0_, 0, 0, 0) = 0. Computing the time derivative of *L*_0_ along the solutions of model (1), we get

$$\begin{array}{c} \begin{array}{cc} \frac{dL_{0}}{dt} & =(1-\frac{S_{0}}{S})[\eta -\beta \rho (1-SD)SI-\mu S]+[\beta \rho (1-SD)SI-(\gamma +\mu )E]\\ & \;+\frac{\left(\gamma +\mu \right)}{\gamma }[\gamma E-(\delta +d+\mu )I]+\frac{\eta \beta \rho (1-SD)}{\delta \mu }\left(1-R_{c}\right)[\delta I-\mu R]. \end{array} \end{array}$$ (4)

Since *S*_0_ = *η*/*μ*, then *η* = *μS*_0_. Thus, after some simplifications, Eq (4) becomes

$$\begin{array}{c} \begin{array}{cc} \frac{dL_{0}}{dt} & =-\mu (S-S_{0})(1-\frac{S_{0}}{S})+\beta \rho \left(1-SD\right)S_{0}I-\frac{\left(\gamma +\mu )(\delta +d+\mu \right)}{\gamma }I\\ & \;+\frac{\eta \beta \rho (1-SD)}{\mu }\left(1-R_{c}\right)I-\frac{\eta \beta \rho (1-SD)}{\delta }\left(1-R_{c}\right)R. \end{array} \end{array}$$ (5)

Substituting for *S*_0_ = *η*/*μ* in the second term of Eq (5) and with further simplifications, we obtain

$$\begin{array}{c} \frac{dL_{0}}{dt}=-\frac{\mu {\left(S-S_{0}\right)}^{2}}{S}-\frac{\left(\gamma +\mu )(\delta +d+\mu \right)}{\gamma }{\left(R_{c}-1\right)}^{2}I-\frac{\eta \beta \rho (1-SD)}{\delta }\left(1-R_{c}\right)R. \end{array}$$

Since $R_{c}<1$, then *dL*_0_/*dt* ≤ 0 for all *S*, *I*, *R* > 0. Also, *dL*_0_/*dt* = 0 when *S*(*t*) = *S*_0_, and *I*(*t*) = *R*(*t*) = 0. Applying the Krasovkii-Lasalle theorem. Suppose that

$$\begin{array}{c} I_{0}=\{\left(S\left(t\right),E\left(t\right),I\left(t\right),R\left(t\right)\right):\frac{dL_{0}}{dt}=0\}, \end{array}$$

and *M*_0_ is the largest invariant subset of $I_{0}$ where all elements in it satisfy *S*(*t*) = *S*_0_ and *I*(*t*) = *R*(*t*) = 0. Then, from the third equation in (1) we get

$$\begin{array}{c} \frac{dI}{dt}=0=\gamma E\;\Rightarrow \;E\left(t\right)=0. \end{array}$$

Hence, *M*_0_ = {*P*_0_} and so the equilibrium *P*_0_ is globally asymptotically stable if $R_{c}<1$.

**Theorem 5**. *The COVID-19 endemic equilibrium point P*_1_ *is globally asymptotically stable if* $R_{c}>1$ .

**Proof**. Consider the Lyapunov function *L*_1_(*S*, *E*, *I*, *R*) as:

$$\begin{array}{c} L_{1}=S_{1}W\left(S/S_{1}\right)+E_{1}W\left(E/E_{1}\right)+\frac{\left(\gamma +\mu \right)}{\gamma }I_{1}W\left(I/I_{1}\right). \end{array}$$

Clearly, *L*_1_(*S*, *E*, *I*, *R*) is positive semi-definite function since *L*_1_ ≥ 0 for all (*S*, *E*, *I*, *R*) ∈ Ω and *L*_1_(*S*_1_, *E*_1_, *I*_1_, *R*_1_) = 0. The time derivative of *L*_1_ along the solutions of model (1) is given by:

$$\begin{array}{c} \begin{array}{cc} \frac{dL_{1}}{dt} & =(1-\frac{S_{1}}{S})\left[\eta -\beta \rho \left(1-SD\right)SI-\mu S\right]+(1-\frac{E_{1}}{E})\left[\beta \rho \left(1-SD\right)SI-\left(\gamma +\mu \right)E\right]\\ & \;+\frac{\left(\gamma +\mu \right)}{\gamma }(1-\frac{I_{1}}{I})\left[\gamma E-\left(\delta +d+\mu \right)I\right]\\ & =(1-\frac{S_{1}}{S})\left[\eta -\mu S\right]+\beta \rho \left(1-SD\right)IS_{1}-\beta \rho \left(1-SD\right)SI\frac{E_{1}}{E}+\left(\gamma +\mu \right)E_{1}\\ & \;-\frac{\left(\gamma +\mu )(\delta +d+\mu \right)}{\gamma }I-\left(\gamma +\mu \right)E\frac{I_{1}}{I}+\frac{\left(\gamma +\mu )(\delta +d+\mu \right)}{\gamma }I_{1}. \end{array} \end{array}$$

From the equilibrium Eq (3), of *P*_1_ we have

$$\begin{array}{cc} \eta & =\beta \rho \left(1-SD\right)S_{1}I_{1}+\mu S_{1},\\ \left(\gamma +\mu \right)E_{1} & =\beta \rho \left(1-SD\right)S_{1}I_{1},\\ \frac{\left(\gamma +\mu )(\delta +d+\mu \right)}{\gamma }I_{1} & =\left(\gamma +\mu \right)E_{1}. \end{array}$$

Then, we get

$$\begin{array}{c} \begin{array}{cc} \frac{dL_{1}}{dt} & =-\frac{\mu {\left(S-S_{1}\right)}^{2}}{S}+3\beta \rho \left(1-SD\right)S_{1}I_{1}-\beta \rho \left(1-SD\right)S_{1}I_{1}\frac{S_{1}}{S}\\ & \;+\beta \rho \left(1-SD\right)IS_{1}-\beta \rho \left(1-SD\right)S_{1}I_{1}\frac{SIE_{1}}{S_{1}I_{1}E}-\left(\gamma +\mu \right)E_{1}\frac{EI_{1}}{E_{1}I}\\ & \;-\frac{\left(\gamma +\mu )(\delta +d+\mu \right)}{\gamma }I. \end{array} \end{array}$$ (6)

We substitute $S_{1}=\eta /\mu R_{c}$ in the fourth term of Eq (6) and note that

$$\begin{array}{c} \left(\gamma +\mu \right)E_{1}\frac{EI_{1}}{E_{1}I}=\beta \rho \left(1-SD\right)S_{1}I_{1}\frac{EI_{1}}{E_{1}I}. \end{array}$$

After simplifying Eq (6), we obtain

$$\begin{array}{c} \frac{dL_{1}}{dt}=-\frac{\mu {\left(S-S_{1}\right)}^{2}}{S}+\beta \rho \left(1-SD\right)S_{1}I_{1}(3-\frac{S_{1}}{S}-\frac{EI_{1}}{E_{1}I}-\frac{SIE_{1}}{S_{1}I_{1}E}). \end{array}$$

Since the arithmetic mean is greater than or equal to the geometric mean, we have

$$\begin{array}{c} 3-\frac{S_{1}}{S}-\frac{EI_{1}}{E_{1}I}-\frac{SIE_{1}}{S_{1}I_{1}E}\leq 0. \end{array}$$

Hence, *dL*_1_/*dt* ≤ 0 for all *S*, *E*, *I* > 0 and *dL*_1_/*dt* = 0 when *S*(*t*) = *S*_1_, *E*(*t*) = *E*_1_, and *I*(*t*) = *I*_1_. Now, applying the Krasovkii-Lasalle theorem, consider the set

$$\begin{array}{c} I_{1}=\{\left(S\left(t\right),E\left(t\right),I\left(t\right),R\left(t\right)\right):\frac{dL_{1}}{dt}=0\}, \end{array}$$

and *M*_1_ is the largest invariant subset of $I_{1}$ where all elements satisfy *S*(*t*) = *S*_1_, *E*(*t*) = *E*_1_, and *I*(*t*) = *I*_1_ it remains to prove that *R*(*t*) = *R*_1_. Assume that (*S*(*t*), *E*(*t*), *I*(*t*), *R*(*t*)) is a solution to model (1) belonging to the set *M*_1_ thus, we have

$$\begin{array}{c} \frac{dR}{dt}=\delta I_{1}-\mu R. \end{array}$$ (7)

Solving Eq (7) by the integrating factor method, we obtain

$$\begin{array}{c} R\left(t\right)=R_{1}+\left(R\left(0\right)-R_{1}\right)e^{-\mu t}. \end{array}$$ (8)

Note that, *δI*_1_/*μ* = *R*_1_. From Eq (8), as time increases *R*(*t*) approaches *R*_1_, that is, $\underset{t\to \infty }{\text{lim}}R(t)\to R_{1}$. The solution (*S*(*t*), *E*(*t*), *I*(*t*), *R*(*t*)) will stay at the set *M*_1_, hence *M*_1_ = {*P*_1_}. Therefore, the equilibrium *P*_1_ is globally asymptotically stable if $R_{c}>1$.

## 4 Numerical analysis

In this section, we validate model (1) by estimating its parameters from fitting the model to the observed actual data. Also, we solve the model numerically and illustrate numerical experiments to show the agreement with the qualitative results. Moreover, we investigate the sensitivity analysis for the control reproduction number. Finally, we analyze numerically different scenarios for the control strategies applied in Saudi Arabia to contain COVID-19, namely, lockdown and social distancing measures.

### 4.1 Model fitting and estimation of parameters

A proposed mathematical model describing a phenomenon is validated by fitting the model with phenomenon existing data. Here, we use the available data on the COVID-19 dashboard of the Saudi Ministry of Health [29] to validate model (1). We consider the active cases of COVID-19 from March 12, 2020, till September 23, 2020, where at this time interval, different levels of lockdown and social distancing were applied in Saudi Arabia.

The fitting of the model is performed by using the nonlinear least-square curve fitting functions in MATLAB. This process is used to estimate the parameters: *β*, *δ*, and *d*. The remaining parameters are either estimated intuitively or obtained from the literature.

The natural death rate, *μ*, is estimated by assuming that the average life span of Saudi individuals is 75 years [30]; thus,

$$\begin{array}{c} \mu =\frac{1}{75\times 365}=3.6529\times 10^{-5}\;\text{per}\;\text{day}. \end{array}$$

From Eq (2), we have *N*(*t*) ≤ *η*/*μ*. Assuming that the population in the absence of the disease is *N*(0) = *η*/*μ*, we estimate the birth rate *η* = 1250. Here, we let *N*(0) = 34218169, the total Saudi population in 2019 [31].

The incubation period is the period of disease development that starts from exposure to the virus till the appearance of disease symptoms [32]. In previous studies, a different incubation period for COVID-19 was introduced. For example, in Hubei province, China, the median incubation period is five days [33]. However, in Saudi Arabia, it is six days [34]. Here, we estimate the parameter *γ* to have the value of 1/6 as in [34].

Next, we estimate the parameters *β*, *δ*, and *d*, the transmission, recovery, and the death due to COVID-19 rates, respectively. We use the nonlinear least square curve fitting technique [14, 35], which gives the best values for the parameters by reducing the error between the COVID-19 reported data points and the numerical solution of the model. In particular, we implement the MATLAB package *lsqcurvefit* with COVID-19 data from March 12, 2020, till April 4, 2020. The fitting is performed on model (9) with no control measures, that is, in the absence of the parameters *ρ* and *SD*, the lockdown and social distance rates, respectively. The model is solved numerically by using the MATLAB package ode45 with initial values: *S*(0) = 34813671, *E*(0) = 105, *I*(0) = 44 and *R*(0) = 1, taken from COVID-19 data.

$$\begin{array}{c} \begin{array}{cc} \frac{dS}{dt} & =\eta -\beta SI-\mu S,\\ \frac{dE}{dt} & =\beta SI-(\gamma +\mu )E,\\ \frac{dI}{dt} & =\gamma E-(\delta +d+\mu )I,\\ \frac{dR}{dt} & =\delta I-\mu R. \end{array} \end{array}$$ (9)

The values of the parameters are summarized in Table 1.

**Table 1:** The description and values of the basic parameters in model (1).

Parameter	Description	Value	Unit	Source
N	Population of Saudi Arabia	34218169	*Individual*	[31]
*η*	Birth rate	1250	*Individual* × *Day*^−1^	Estimated
*μ*	Natural death rate	3.6529 × 10^−5^	*Day* ^−1^	Estimated
*β*	Transmission rate	1.0063 × 10^−7^	(*Individual* × *Day*)^−1^	Fitted
*γ*	Incubation rate	1/6	*Day* ^−1^	[34]
*δ*	Recovery rate	3.2772 × 10^−1^	*Day* ^−1^	Fitted
*d*	Death rate due to COVID-19	2.3724 × 10^−1^	*Day* ^−1^	Fitted

**Fig 2:**
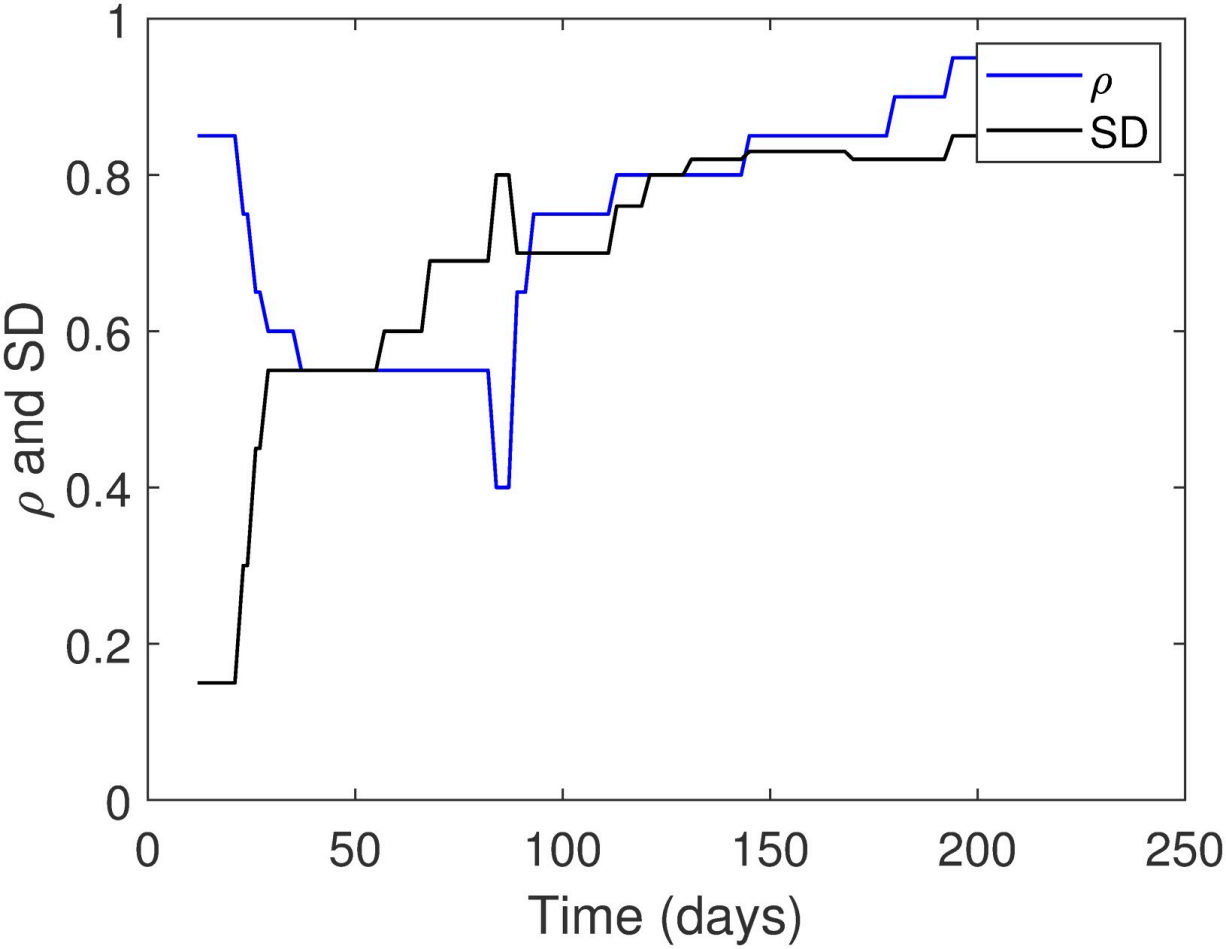
Time variation of the parameters: The lockdown, *ρ*, and the social distancing, *SD*.

Finally, we estimate the values of the rates of lockdown, *ρ*, and social distancing, *SD*, by dividing the entire period from March 12, 2020, till September 23, 2020, into seven phases. Some phases are divided into more than one period based on the different implementation of the preventive measures (see subsection 4.4). Table 2 presents the values of *ρ* and *SD* in all phases, which is also presented graphically in Fig 2. Note that when the value of *SD* is closer to unity, then this implies that social distancing is being practiced more. On the contrary, if the value of *ρ* is closer to unity, it indicates that lockdown measures are executed less. Also, in Table 2, we show the computed value of $R_{c}$ in each phase. In Subsection 4.4, we demonstrate in more detail the strategies applied in each phase to control the COVID-19 pandemic in Saudi Arabia.

**Fig 3:**
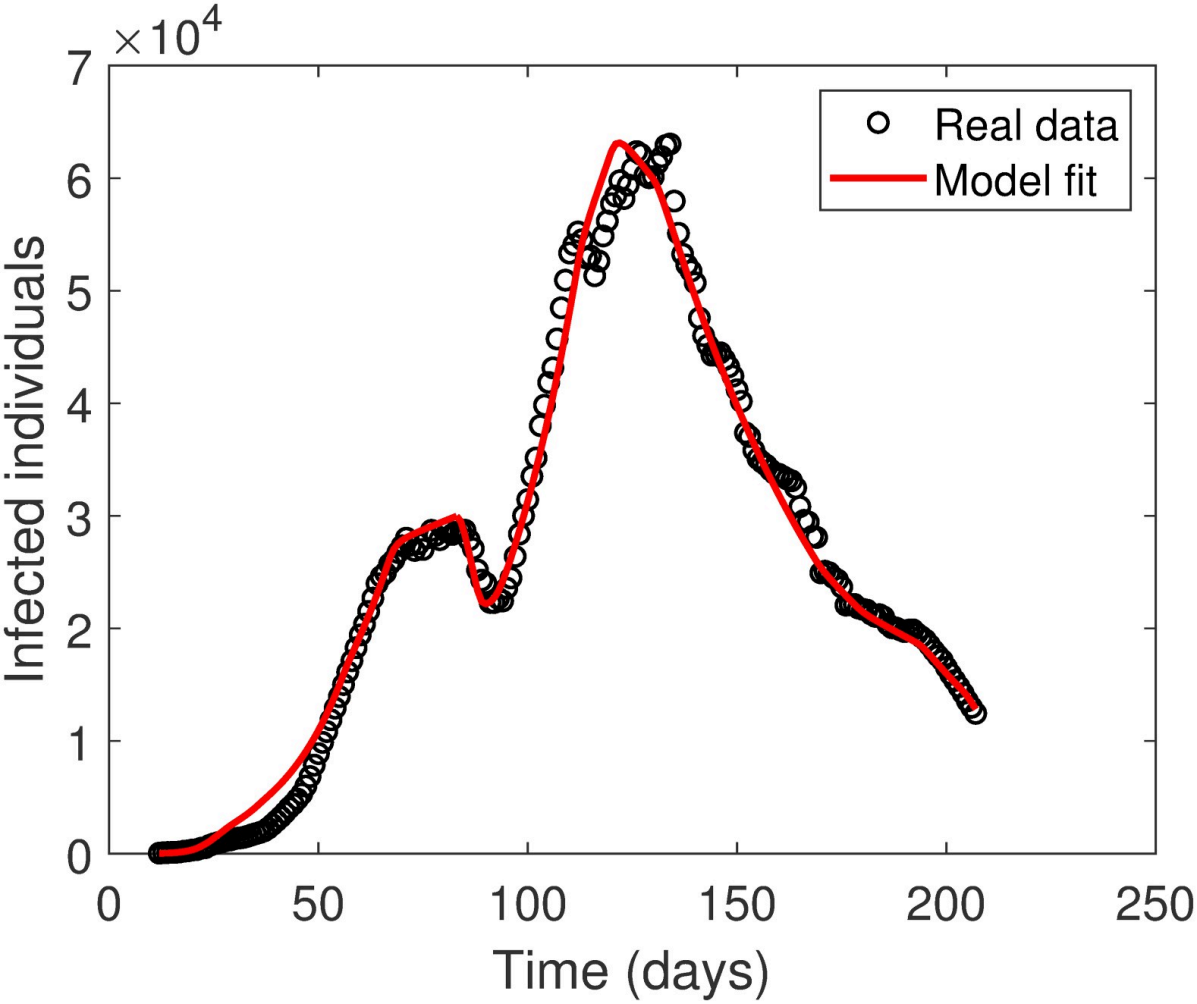
Fitting model (1) for COVID-19 active cases in Saudi Arabia from March 12, 2020, till September 23, 2020.

In Fig 3, we simulate numerically model (1) to illustrate the time variation of the infected compartment (active cases) from March 12, 2020, till September 23, 2020. The curve of the infected compartment is demonstrated along side with the real data for COVID-19 active cases in Saudi Arabia. From the figure, we conclude that the values of the parameters in Tables 1 and 2 are good estimation for fitting model (1) to the actual data.

**Table 2:** Estimated values for *ρ* and *SD* in model (1) with corresponding values of $R_{c}$.

Phase	Time	*ρ*	*SD*	$R_{c}$
Phase 1	*t*1 = 12: 22	0.85	0.15	4.4025
Phase 2	*t*2 = 22: 25	0.75	0.30	3.1990
	*t*3 = 25: 28	0.65	0.45	2.1784
	*t*4 = 28: 36	0.60	0.55	1.6452
Phase 3	*t*5 = 36: 56	0.55	0.55	1.5081
Phase 4	*t*6 = 56: 67	0.55	0.60	1.3405
	*t*7 = 67: 83	0.55	0.69	1.0389
Phase 5	*t*8 = 83: 88	0.40	0.80	0.4875
Phase 6	*t*9 = 88: 92	0.65	0.70	1.1882
	*t*10 = 92: 112	0.75	0.70	1.3710
Phase 7	*t*11 = 112: 120	0.80	0.76	1.1699
	*t*12 = 120: 130	0.80	0.80	0.9749
	*t*13 = 130: 144	0.80	0.82	0.8774
	*t*14 = 144: 169	0.85	0.83	0.8805
	*t*15 = 169: 179	0.85	0.82	0.9323
	*t*16 = 179: 193	0.90	0.82	0.9871
	*t*17 = 193: 204	0.95	0.85	0.8683
	*t*18 = 204: 207	0.95	0.88	0.6946

### 4.2 Numerical experiments

We perform numerical simulations for the model (1) for different initial values as we aim to show the consistency between the numerical solution and the qualitative analysis of the model presented in Section 3. The analytical results state that the solution curves approach *P*_0_ if $R_{c}<1$ and *P*_1_ if $R_{c}>1$. To proceed with the numerical analysis, we first re-scale the state variables in the model. Let

$$\begin{array}{c} S=\bar{S}N,\;E=\bar{E}N,\;I=\bar{I}N,\;R=\bar{R}N. \end{array}$$ (10)

Substituting (10) into model (1), we obtain the re-scaled model (omitting the bar onward):

$$\begin{array}{c} \begin{array}{cc} \frac{dS}{dt} & =\mu -\beta \rho \left(1-SD\right)\frac{\eta }{\mu }SI-\mu S,\\ \frac{dE}{dt} & =\beta \rho \left(1-SD\right)\frac{\eta }{\mu }SI-\left(\gamma +\mu \right)E,\\ \frac{dI}{dt} & =\gamma E-(\delta +d+\mu )I,\\ \frac{dR}{dt} & =\delta I-\mu R. \end{array} \end{array}$$ (11)

Here, we have used *N* = *η*/*μ*, the limiting value of *N* as time increases.

The model (11) is solved numerically using ode45, with the following different initial conditions:

IC1: *S*(0) = 0.8, *E*(0) = 0.1, *I*(0) = 0.05, *R*(0) = 0.01,
IC2: *S*(0) = 0.6, *E*(0) = 0.2, *I*(0) = 0.15, *R*(0) = 0.07,
IC3: *S*(0) = 0.4, *E*(0) = 0.3, *I*(0) = 0.2, *R*(0) = 0.1.

The initial conditions are chosen from the set $\bar{\Omega }=\left\{\left(S,E,I,R\right):0<S+E+I+R\leq 1\right\}$. The model’s parameters are chosen to satisfy the stability conditions for each equilibrium in the qualitative analysis.

**Fig 4:**
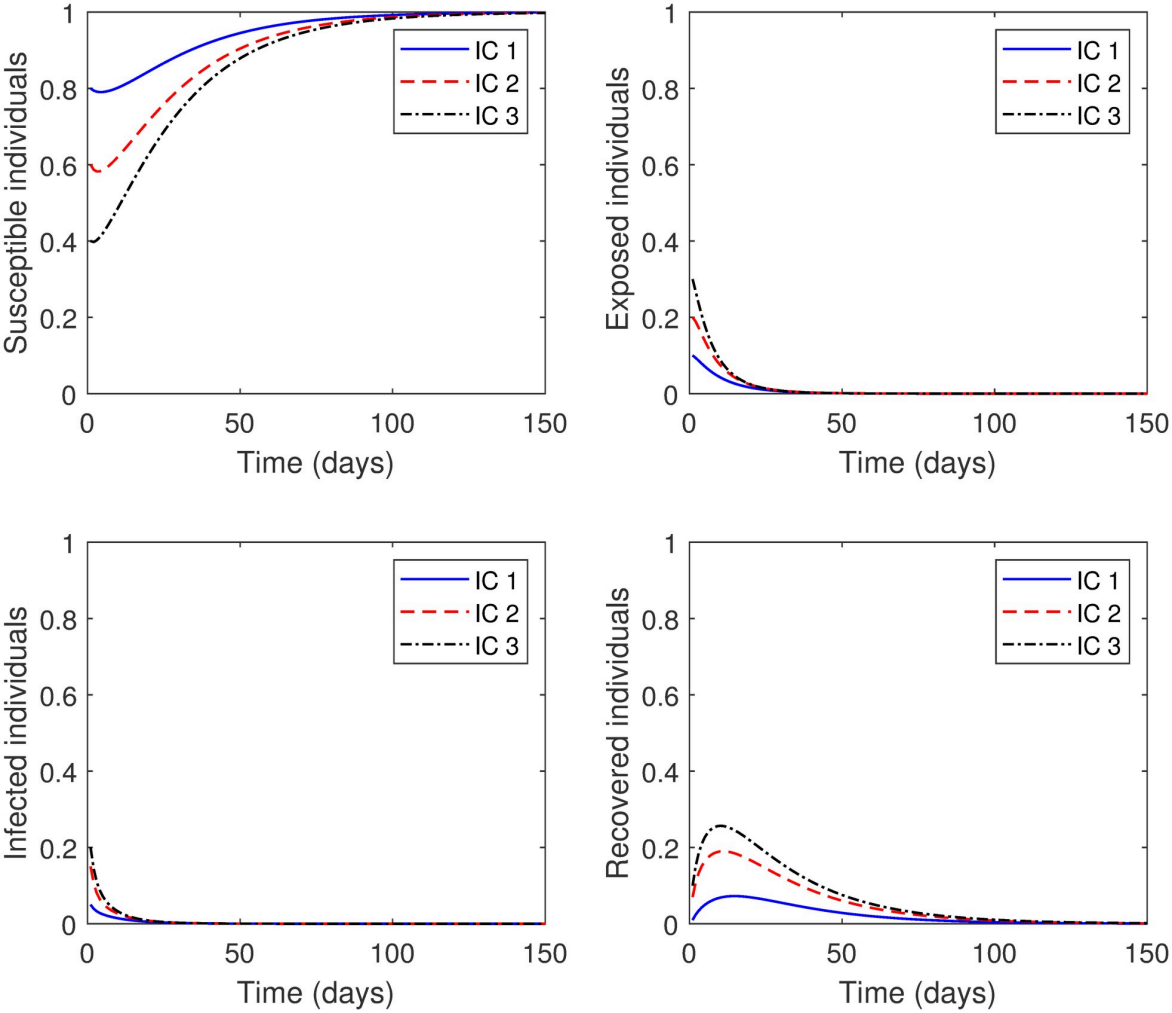
Numerical solution of model (1) with different initial conditions for $R_{c}<1$.

**Fig 5:**
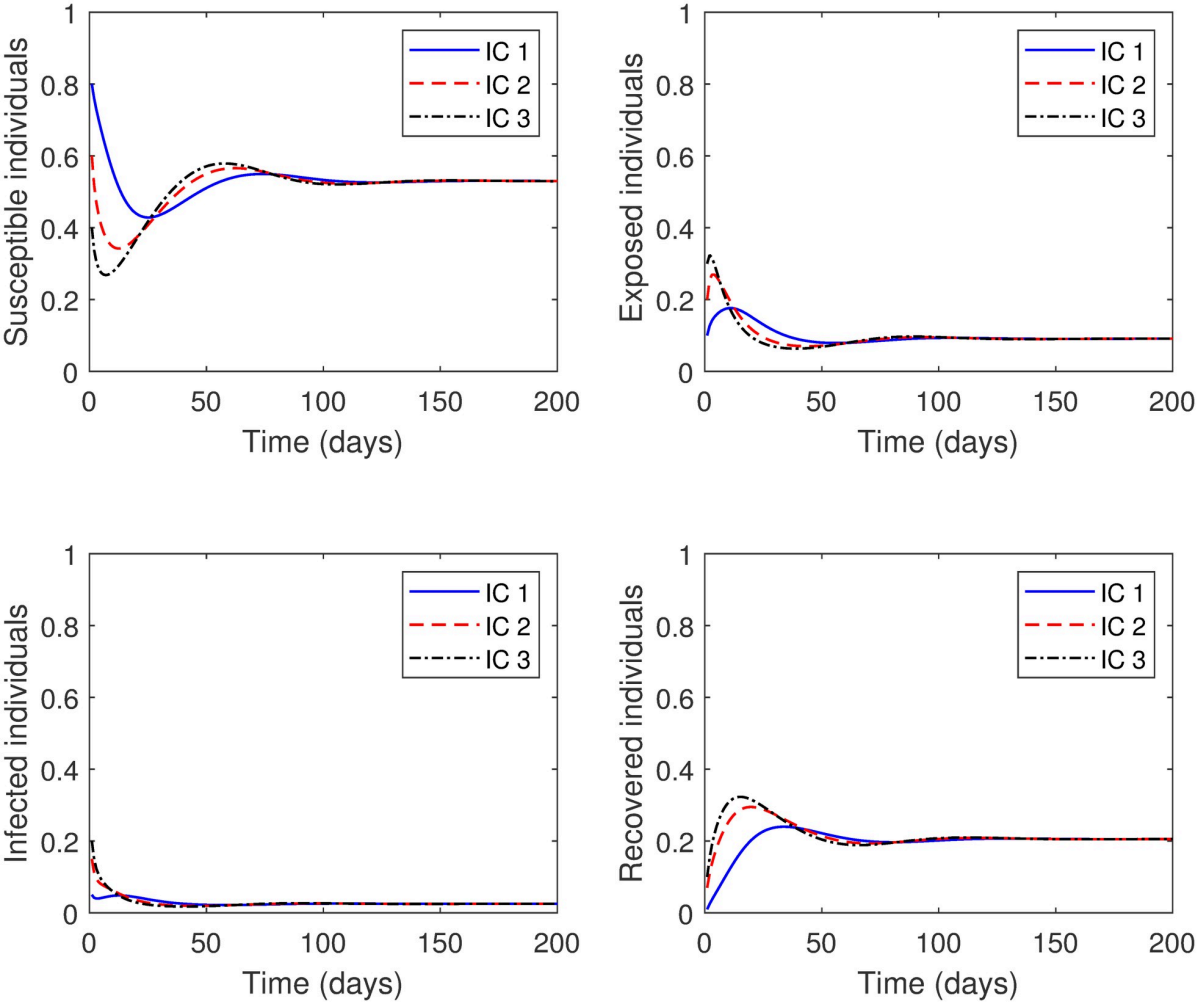
Numerical solution of model (1) with different initial conditions for $R_{c}>1$.

**Experiment 1**: Let the parameters in model (11) take the following values: *μ* = 0.04, *η* = 1250, *γ* = 0.167, *β* = 1.0063 × 10^−4^, *δ* = 3.2772 × 10^−1^, *d* = 2.3724 × 10^−1^, *ρ* = 0.5, and *SD* = 0.75. Here, $R_{c}=0.5242<1$. Thus, the solution curves of the model should approach the COVID-19 free equilibrium point *P*_0_ = (1, 0, 0, 0). This is apparent in Fig 4, we see that for different initial conditions, the numerical solutions eventually reach *P*_0_. Consequently, the community will be free of COVID-19 disease.
**Experiment 2**: Let the parameters in model (11) take the following values: *μ* = 0.04, *η* = 1250, *γ* = 0.167, *β* = 1.0063 × 10^−4^, *δ* = 3.2772 × 10^−1^, *d* = 2.3724 × 10^−1^, *ρ* = 0.9, and *SD* = 0.5. Here, we increased the value of the parameter *ρ* and decreased the value of the parameter *SD*. In other words, the control measures of both lockdown and social distancing are lessened. In this case, $R_{c}=1.8872>1$. As a result, we expect the solution curves of the model reach the COVID-19 endemic equilibrium point *P*_1_ = (0.5295, 0.0910, 0.0251, 0.2056). This is illustrated in Fig 5, where see that for different initial conditions, the numerical solutions eventually tend to *P*_1_. This means that COVID-19 disease remains in the community at a specific value.

From the above experiments, we conclude that the numerical results agree with the qualitative results.

### 4.3 Sensitivity analysis for $R_{c}$

We analyze the sensitivity of the control reproduction number for model (1). This analysis determines which parameters are most effective in $R_{c}$ and control the COVID-19 disease by making $R_{c}<1$. The control reproduction number $R_{c}$ depends on all parameters of model (1). We investigate the sensitivity of $R_{c}$ analytically by evaluating $\partial R_{c}/\partial P$, where P=(η,β,ρ,SD,γ,δ,d,μ). The rate of change of $R_{c}$ with respect to one parameter at a time is the following:

$$\begin{array}{c} \begin{array}{cc} \frac{\partial R_{c}}{\partial \eta } & =\frac{\beta \rho (1-SD)\gamma }{\mu (\gamma +\mu )(\delta +d+\mu )}>0,\\ \frac{\partial R_{c}}{\partial \beta } & =\frac{\rho (1-SD)\eta \gamma }{\mu (\gamma +\mu )(\delta +d+\mu )}>0,\\ \frac{\partial R_{c}}{\partial \rho } & =\frac{\beta (1-SD)\eta \gamma }{\mu (\gamma +\mu )(\delta +d+\mu )}>0,\\ \frac{\partial R_{c}}{\partial SD} & =\frac{-\beta \rho \eta \gamma }{\mu (\gamma +\mu )(\delta +d+\mu )}<0,\\ \frac{\partial R_{c}}{\partial \gamma } & =\frac{\beta \rho (1-SD)\eta }{{\left(\gamma +\mu \right)}^{2}\left(\delta +d+\mu \right)}>0,\\ \frac{\partial R_{c}}{\partial \delta } & =\frac{-\beta \rho (1-SD)\eta \gamma }{\mu \left(\gamma +\mu \right){\left(\delta +d+\mu \right)}^{2}}<0,\\ \frac{\partial R_{c}}{\partial d} & =\frac{-\beta \rho (1-SD)\eta \gamma }{\mu \left(\gamma +\mu \right){\left(\delta +d+\mu \right)}^{2}}<0,\\ \frac{\partial R_{c}}{\partial \mu } & =\frac{-\beta \rho \left(1-SD\right)\eta \gamma (3\mu^{2}+2\mu \left(\gamma +\delta +d\right)+\gamma \left(\delta +d\right))}{\mu^{2}{\left(\gamma +\mu \right)}^{2}{\left(\delta +d+\mu \right)}^{2}}<0. \end{array} \end{array}$$ (12)

**Fig 6:**
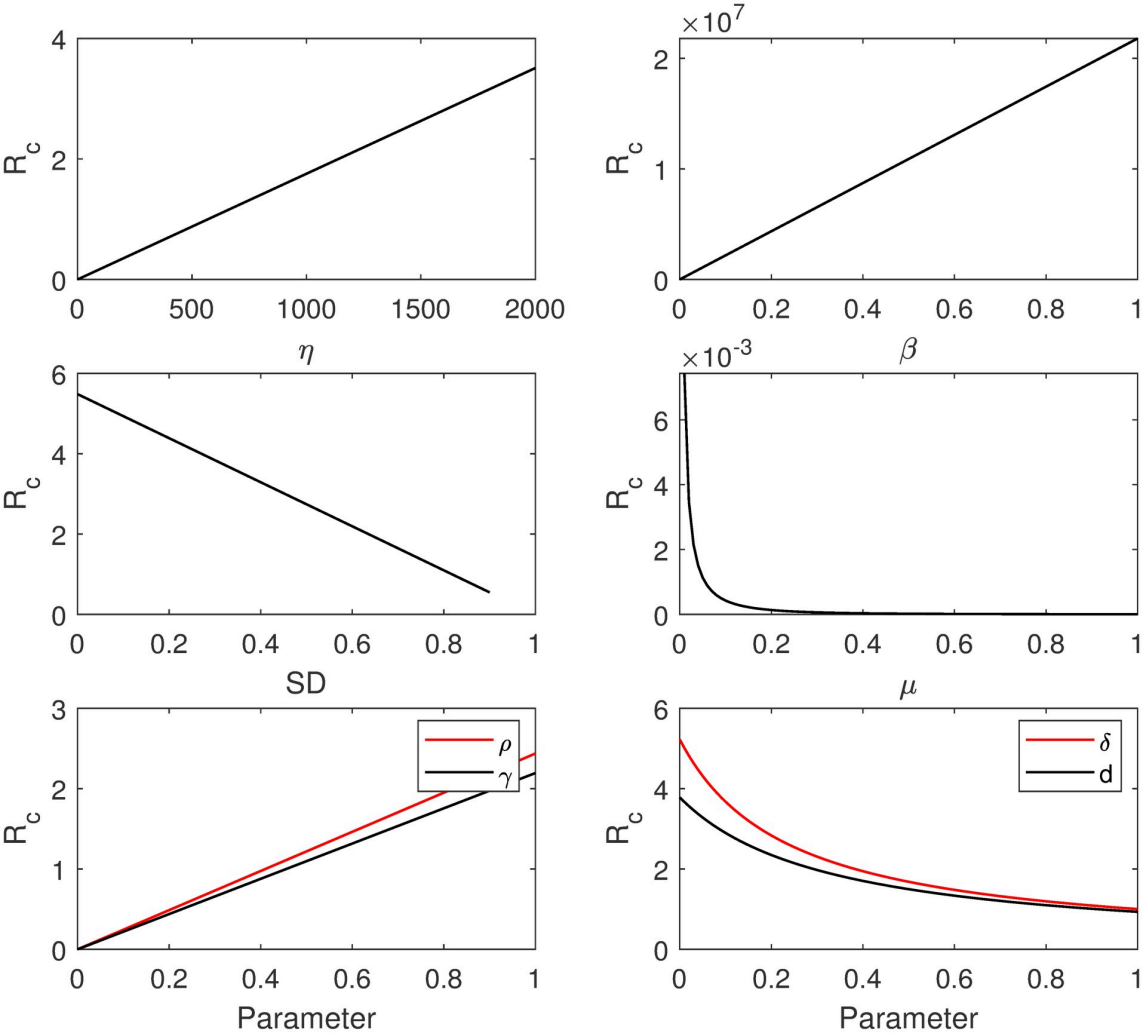
The sensitivity of $R_{c}$ with respect to the parameters of model (1).

Eq (12) shows that $R_{c}$ decreases when *SD*, *δ*, *d*, and *μ* increase. On the contrary, $R_{c}$ increases when *η*, *β*, *ρ*, and *γ* increase. Note that the increase in the value of *ρ* means that lockdown application is decreased. This result is illustrated in Fig 6. In addition, we compute the normalized sensitivity index (elasticity) of $R_{c}$ with respect to the models’ parameters P, which is defined as the following [32]:

$$\begin{array}{c} \Gamma_{R_{c}}^{P}=\frac{\partial R_{c}}{\partial P}\frac{P}{R_{c}}. \end{array}$$

By applying the formula, we get

$$\begin{array}{c} \begin{array}{cc} \Gamma_{R_{c}}^{\eta } & =\frac{\beta \rho (1-SD)\gamma }{\mu (\gamma +\mu )(\delta +d+\mu )}\frac{\eta }{R_{c}}=1,\\ \Gamma_{R_{c}}^{\beta } & =\frac{\rho (1-SD)\eta \gamma }{\mu (\gamma +\mu )(\delta +d+\mu )}\frac{\beta }{R_{c}}=1,\\ \Gamma_{R_{c}}^{\rho } & =\frac{\beta (1-SD)\eta \gamma }{\mu (\gamma +\mu )(\delta +d+\mu )}\frac{\rho }{R_{c}}=1,\\ \Gamma_{R_{c}}^{SD} & =\frac{-SD}{1-SD},\\ \Gamma_{R_{c}}^{\gamma } & =\frac{\mu }{\gamma +\mu },\\ \Gamma_{R_{c}}^{\delta } & =\frac{-\delta }{\left(\delta +d+\mu \right)},\\ \Gamma_{R_{c}}^{d} & =\frac{-d}{\left(\delta +d+\mu \right)},\\ \Gamma_{R_{c}}^{\mu } & =\frac{-(3\mu^{2}+2\mu \left(\gamma +\delta +d\right)+\gamma \left(\delta +d\right))}{\left(\gamma +\mu )(\delta +d+\mu \right)}. \end{array} \end{array}$$

Table 3 shows the elasticity values of $R_{c}$ with respect to the models’ parameters P, where the values of the parameters are given in Table 1. The positive (negative) signs of the values in Table 3 indicate increasing (decreasing) values of $R_{c}$ with respect to P. For instance, $\Gamma_{R_{c}}^{\gamma }=0.00021869$ means that an increase in *γ* by 1% will increase $R_{c}$ by 0.00021%. Whereas, $\Gamma_{R_{c}}^{\delta }=-0.9440$ means that an increase in *δ* by 1% will decrease $R_{c}$ by 0.9440%. The remaining parameters in Table 3 have similar interpretations.

**Table 3:** The sensitivity index of $R_{c}$ with respect to the parameters P of model (1).

Parameter P	Value	Sensitivity index $\Gamma_{R_{c}}^{P}$
*η*	1250	1
*β*	1.0063 × 10^−7^	1
*ρ*	-	1
*γ*	0.167	0.00021869
*δ*	3.2772 × 10^−1^	−0.9440
*d*	2.3724 × 10^−1^	−0.0556
*μ*	3.653 × 10^−5^	−1.0007
*SD*	0.10	−0.1111
	0.20	−0.2500
	0.30	−0.4286
	0.40	−0.6667
	0.45	−0.8182
	0.50	−1
	0.60	−1.5000
	0.70	−2.3333
	0.80	−4
	0.90	−9
	0.95	−19
	0.99	−99

In particular, Table 3 illustrates the effect of the control parameters *ρ* and *SD* on $R_{c}$. Reducing the value of *ρ*, which means increasing lockdown application, helps in lowering the value of $R_{c}$. Since a 1% increase in *ρ* corresponds to a 1% increase in $R_{c}$ and vise versa. Also, increasing the application of social distancing *SD* helps in reducing the value of $R_{c}$, especially if the value of *SD* = 0.50 or more. For example, if the value of social distancing application is *SD* = 0.95, then a 1% increase in *SD* leads to a decrease in $R_{c}$ by 19%.

### 4.4 Scenarios for lockdown and social distancing

Saudi Arabia has implemented various policies to limit COVID-19 disease. The main strategies were enforcement of lockdown and social distancing. Fig 3 demonstrates the actual data of the active cases from March 12, 2020, till September 23, 2020, due to the execution of these strategies. Also, the figure shows, for the same period, the fitting curve from model (1) for the active cases (infected individuals). We will use the model to explore different scenarios for lockdown (*ρ*) and social distancing (*SD*) on the expected number of active cases. The model is solved numerically with the initial values: *S*(0) = 34813671, *E*(0) = 105, *I*(0) = 44, *R*(0) = 1, and parameters’ values in Table 1. The scenarios are produced according to the changes in the values of *ρ* ∈ (0, 1] and *SD* ∈ [0, 1). Recall that extreme lockdown measures are performed when *ρ* approaches the value zero, whereas social distancing is highly achieved when *SD* is near one.

We divided the entire period from March 12, 2020, till September 23, 2020, into seven phases, illustrated in Table 2, and these phases are as follows:

**Phase 1**: [5, 36–41] The time interval in this phase is *t*1 = [12, 22] days, where the estimated values of *ρ* and *SD* from the fitting are 0.85 and 0.15, respectively. At this phase, the government of Saudi Arabia has imposed some decisions such as entry suspension to Saudi Arabia for individuals holding tourist visas and arriving from countries where the spread of the COVID-19 poses a danger; entry suspension to Saudi Arabia for Umrah, and visiting the Prophet’s Mosque; suspension of Friday prayers and congregation except for the Two Holy Mosques; closing schools and universities; suspension of attending workplaces; and suspending all domestic flights, buses, taxis, and trains in all cities. The decisions in this phase are carried out to Phase 5. While, the decisions related to Umrah and entering Saudi Arabia are carried out to Phase 7.
**Phase 2**: [42–44] The time interval in this phase is divided into three periods of time *t*2 = [22, 25] days, *t*3 = [25, 28] days, and *t*4 = [28, 36] days. The estimated fitting values of *ρ* in *t*2, *t*3 and *t*4 are 0.75, 0.63 and 0.60, respectively. As for *SD*, they are 0.30, 0.45, and 0.55 in the three periods, respectively. At this phase, the government of Saudi Arabia imposed partial lockdown from 7 P.M. to 6 A.M. (11-hours) in all cities at *t*2. In period *t*3, they prevented movement between regions of Saudi Arabia, prohibited entry and exit from Riyadh, Makkah, and Medina cities, and imposed partial lockdown from 3 P.M. to 6 A.M. (16-hours) in Riyadh, Makkah, and Medina. At the same time, partial lockdown continued in the other cities from 7 P.M. to 6 A.M. As for period *t*4; They prohibited entry and exit from Jeddah and imposed partial lockdown from 3 P.M. to 6 A.M. in Jeddah while partial lockdown continued in the other cities from 7 P.M. to 6 A.M. The decision to prevent movement between regions of Saudi Arabia is carried out to Phase 5.
**Phase 3**: [45, 46] The time interval in this phase is *t*5 = [36, 56] days, where the estimated values of *ρ* and *SD* from the fitting are equal to 0.55. At this phase, the government of Saudi Arabia imposed a complete lockdown (24-hour) in Riyadh, Tabuk, Dammam, Dhahran, and Al-Hofuf cities, as well as throughout the governorates: Jeddah, Taif, Qatif, and Khobar. However, partial lockdown continues in the other cities from 7 P.M. to 6 A.M. (11-hours).
**Phase 4**: [47, 48] The time interval in this phase is divided into two periods of time *t*6 = [56, 67] days, and *t*7 = [67, 83] days. The estimated value of *ρ* from the fitting is 0.55 for the two periods. However, the values of *SD* in *t*6 and *t*7 are 0.60 and 0.69, respectively. At this phase, the government of Saudi Arabia imposed partial lockdown from 5 P.M. to 9 A.M. (16-hours) in all cities, except for Makkah, which was kept under a complete lockdown.
**Phase 5**: [48] The time interval in this phase is *t*8 = [83, 88] days, where the estimated values of *ρ* and *SD* from the fitting are 0.40 and 0.80, respectively. At this phase, the government of Saudi Arabia imposed a complete lockdown (24-hours) in all cities.
**Phase 6**: [49] The time interval in this phase is divided into two periods of time *t*9 = [88, 92] days, and *t*10 = [92, 112] days. The estimated value of *SD* from the fitting is 0.70. As for *ρ*, the values are 0.65 and 0.75 in *t*9 and *t*10, respectively. At this phase, in period *t*9, the government of Saudi Arabia imposed partial lockdown from 3 P.M. to 6 A.M. (15-hours) in all cities, except for Makkah, which was kept under a complete lockdown. However, they allowed some economic and commercial activities and removed the suspension of travel between regions of Saudi Arabia. In period *t*10, they imposed partial lockdown from 8 P.M. to 6 A.M. (10-hours) in all cities, except for Makkah, a partial lockdown was from 3 P.M. to 6 A.M. (15-hours). However, they allowed employees attendance of ministries, government agencies, and private sector companies; Friday and congregational prayers in mosques, and the suspension of domestic flights was removed.
**Phase 7**: [50–52] The time interval in this phase is [112, 207] days, which is divided into eight periods from *t*11 to *t*18 (see Table 2). At this phase, the government of Saudi Arabia allowed a return to normalcy but with some preventive measures such as social distancing, sanitization, and imperative wearing of a face mask. The return to work was permitted to 75% of employees in all cities. The opening of malls, parks, and sports clubs was announced. However, the suspension of overseas Umrah, international flights, and entry and exit across land and sea borders were still imposed.

We begin the investigation by considering two cases. In the first case, the lockdown and social distancing levels are varied for one phase only, while in the rest phases, *ρ* and *SD* are kept as estimated in the fitting (see Table 2). We focus on varying Phase 1 through Phase 6, leaving Phase 7 out of the analysis since the data of active cases in this phase began to decline with a high level of social distancing (0.76–0.88) and ease measures of lockdown (0.80–0.95). In the second case, the analysis is carried out for all the phases at once. In all cases, we compare the resulting number of active cases with the actual number of active cases in the fitting. In particular, we compute the percentage change between the peak values of the active cases in the scenario and the fitting. The peak of active cases in the fitting is 63,130 cases and occurs on day 122. The positive sign in the percentage value indicates an increase, and the negative sign indicates a decrease in active cases compared to the actual data.

**First Case**: Different scenarios are presented when varying *ρ* and *SD* in a selected phase while keeping their values for the other phases as in Table 2. The following eight scenarios will be discussed for each phase separately.

1^*st*^ **scenario**: This scenario analyzes the effect of no lockdown enforcement (*ρ* = 1), while keeping the social distancing (*SD*) value the same as the fitting value (see Table 2).
2^*nd*^ **scenario**: This scenario analyzes the effect of no lockdown enforcement (*ρ* = 1) with increased values of social distancing (*SD*).
3^*rd*^ **scenario**: This scenario analyzes the effect of increasing lockdown while keeping the social distancing (*SD*) value the same as the fitting value.
4^*th*^ **scenario**: This scenario analyzes the effect of increasing the social distancing (*SD*) value while keeping the lockdown value the same as the fitting value.
5^*th*^ **scenario**: This scenario analyzes the effect of decreasing lockdown while keeping the social distancing (*SD*) value the same as the fitting value.
6^*th*^ **scenario**: This scenario analyzes the effect of decreasing social distancing (*SD*) value while keeping the lockdown value the same as the fitting value.
7^*th*^ **scenario**: This scenario analyzes the effect of increasing lockdown and social distancing (*SD*) values simultaneously.
8^*th*^ **scenario**: This scenario analyzes the effect of decreasing lockdown value but increasing the value of social distancing (*SD*).

In addition to the above scenarios, we will discuss more scenarios in Phase 2, Phase 3, Phase 5, and Phase 6, as follows:

9^*th*^ **scenario**: This scenario analyzes the effect of the continued lockdown level from the previous phase while the social distancing (*SD*) value is kept the same as the fitting value. For instance, when applying this scenario to Phase 2, the value of lockdown equals the value of lockdown in Phase 1.
10^*th*^ **scenario**: This scenario analyzes the effect of continued lockdown level from the previous phase with increasing values of social distancing (*SD*).

**Fig 7:**
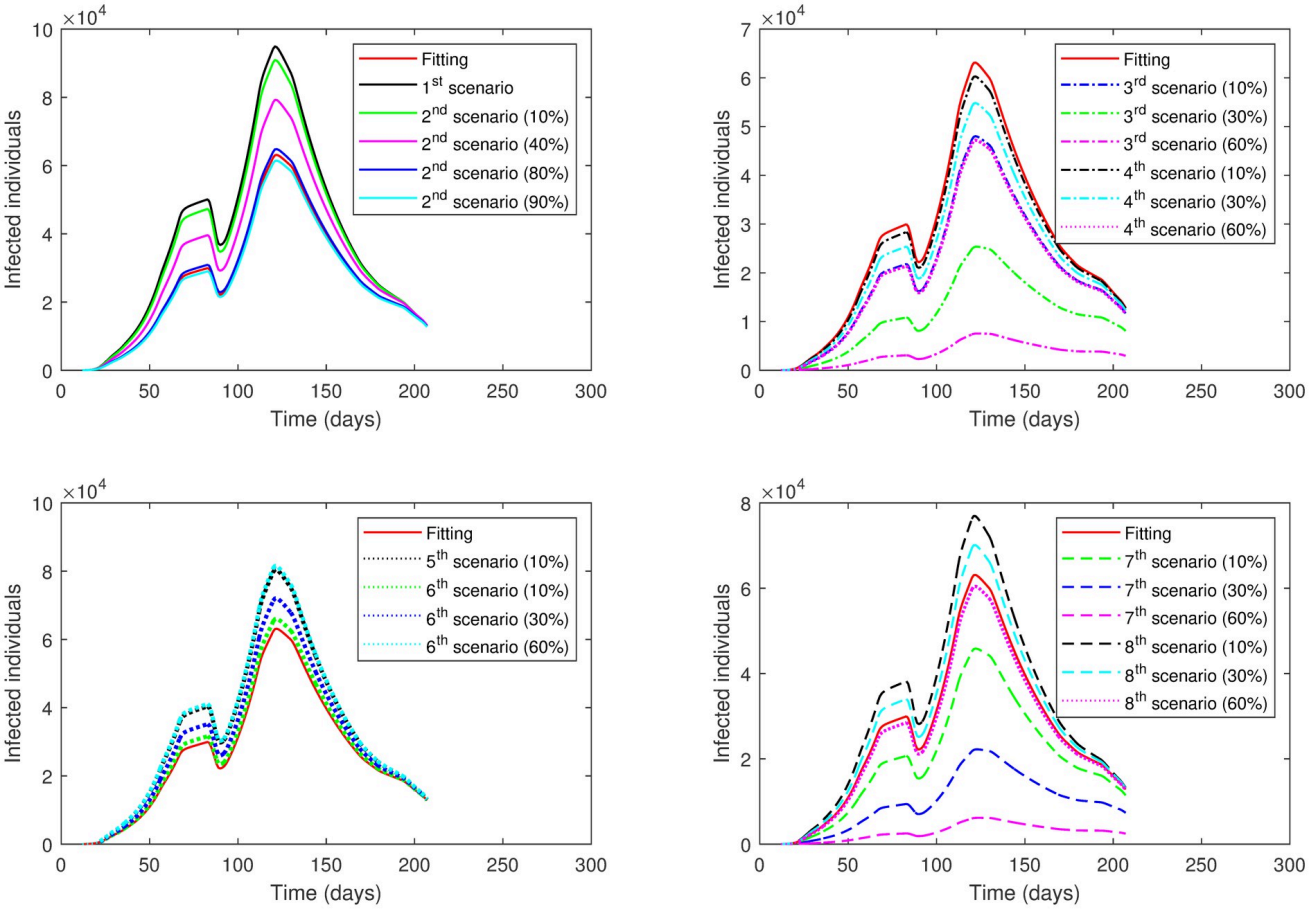
The numerical solution of model (1) for infected compartment vs. time with different scenarios of Phase 1 in the First Case: 1^*st*^ scenario, no lockdown is implemented; 2^*nd*^ scenario, no lockdown while *SD* increases; 3^*rd*^ scenario, lockdown increases; 4^*th*^ scenario, *SD* increases; 5^*th*^ scenario, lockdown decreases; 6^*th*^ scenario, *SD* decreases; 7^*th*^ scenario, lockdown and *SD* increases; 8^*th*^ scenario, lockdown decreases and *SD* increases.

The results of applying the scenarios (1^*st*^—8^*th*^) to **Phase 1** is presented in Table 4 and illustrated in Fig 7. Note that the values of *ρ* and *SD* are varied only in the selected phase (here Phase 1), while in the other phases, they are kept as estimated in the fitting.

**Table 4:** Results of different scenarios for Phase 1.

Scenarios	*ρ*	*SD*	Peak(day)	Active Cases	Change Percentage	Notes
	0.85	0.15	122	63,130	-	The fitting value
1^*st*^ scenario	1	0.15	121	94,900	+50.32%	No lockdown enforcement
2^*nd*^ scenario	1	0.165	121	90,920	+44.02%	Increase *SD* by 10%
	1	0.18	121	86,980	+37.77%	Increase *SD* by 20%
	1	0.195	121	83,090	+31.61%	Increase *SD* by 30%
	1	0.21	121	79,260	+25.55%	Increase *SD* by 40%
	1	0.225	121,122	75,510	+19.61%	Increase *SD* by 50%
	1	0.24	122	71,860	+13.82%	Increase *SD* by 60%
	1	0.255	122	68,300	+8.18%	Increase *SD* by 70%
	1	0.27	122	64,830	+2.69%	Increase *SD* by 80%
	1	0.285	122	61,450	−2.66%	Increase *SD* by 90%
3^*rd*^ scenario	0.765	0.15	122	48,020	−23.93%	Increase lockdown by 10%
	0.68	0.15	122	35,430	−43.87%	Increase lockdown by 20%
	0.595	0.15	123	25,360	−59.82%	Increase lockdown by 30%
	0.51	0.15	123	17,580	−72.15%	Increase lockdown by 40%
	0.425	0.15	123,124	11,760	−81.37%	Increase lockdown by 50%
	0.34	0.15	124	7,558	−88.02%	Increase lockdown by 60%
4^*th*^ scenario	0.85	0.165	122	60,290	−4.49%	Increase *SD* by 10%
	0.85	0.18	122	57,520	−8.88%	Increase *SD* by 20%
	0.85	0.195	122	54,830	−13.14%	Increase *SD* by 30%
	0.85	0.21	122	52,210	−17.29%	Increase *SD* by 40%
	0.85	0.225	122	49,670	−21.32%	Increase *SD* by 50%
	0.85	0.24	122	47,210	−25.21%	Increase *SD* by 60%
5^*th*^ scenario	0.935	0.15	121	80,470	+27.47%	Decrease lockdown by 10%
6^*th*^ scenario	0.85	0.135	122	66,030	+4.59%	Decrease *SD* by 10%
	0.85	0.12	122	69,010	+9.31%	Decrease *SD* by 20%
	0.85	0.105	122	72,040	+14.11%	Decrease *SD* by 30%
	0.85	0.09	121,122	75,140	+19.02%	Decrease *SD* by 40%
	0.85	0.075	121	78,320	+24.06%	Decrease *SD* by 50%
	0.85	0.06	121	81,550	+29.17%	Decrease *SD* by 60%
7^*th*^ scenario	0.765	0.165	122	45,850	−27.37%	Increase lockdown and *SD* by 10%
	0.68	0.18	122,123	32,340	−48.77%	Increase lockdown and *SD* by 20%
	0.595	0.195	123	22,230	−64.78%	Increase lockdown and *SD* by 30%
	0.51	0.21	123	14,900	−76.39%	Increase lockdown and *SD* by 40%
	0.425	0.225	123,124	9,729	−84.58%	Increase lockdown and *SD* by 50%
	0.34	0.24	124	6,179	−90.21%	Increase lockdown and *SD* by 60%
8^*th*^ scenario	0.935	0.165	121	76,930	+21.85%	Decrease lockdown and increase *SD* by 10%
	0.935	0.18	122	73,480	+16.39%	Decrease lockdown and increase *SD* by 20%
	0.935	0.195	122	70,110	+11.05%	Decrease lockdown and increase *SD* by 30%
	0.935	0.21	122	66,820	+5.84%	Decrease lockdown and increase *SD* by 40%
	0.935	0.225	122	63,610	+0.76%	Decrease lockdown and increase *SD* by 50%
	0.935	0.24	122	60,470	−4.21%	Decrease lockdown and increase *SD* by 60%
	0.935	0.255	122	57,430	−9.02%	Decrease lockdown and increase *SD* by 70%
	0.935	0.27	122	54,480	−13.70%	Decrease lockdown and increase *SD* by 80%
	0.935	0.285	122	51,610	−18.24%	Decrease lockdown and increase *SD* by 90%

Phase 2 has three time periods with different decisions; thus, we added two more scenarios as follows:

11^*th*^ **scenario**: This scenario analyzes the effect of the continued lockdown level in all Phase 2, as in period *t*2, while keeping the social distancing (*SD*) value the same as the fitting value.
12^*th*^ **scenario**: This scenario analyzes the effect of the continued lockdown level in all Phase 2, as in period *t*2, while increasing the social distancing (*SD*) value.

**Fig 8:**
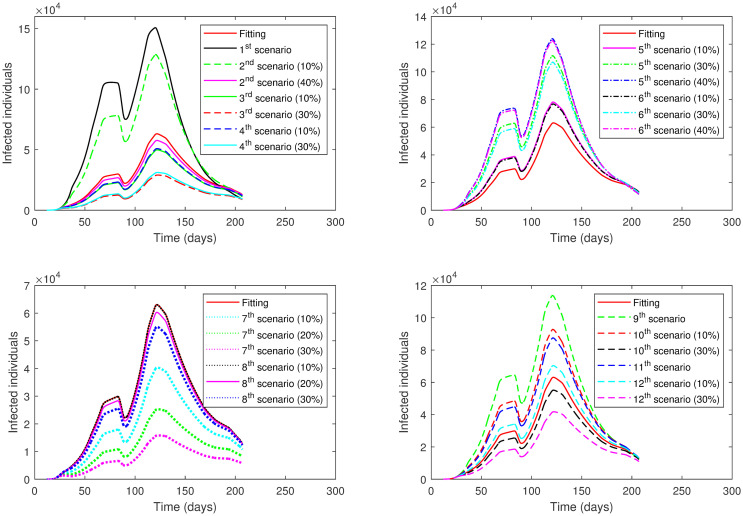
The numerical solution of model (1) for infected compartment vs. time with different scenarios of Phase 2 in the First Case: 1^*st*^ scenario, no lockdown is implemented; 2^*nd*^ scenario, no lockdown while *SD* increases; 3^*rd*^ scenario, lockdown increases; 4^*th*^ scenario, *SD* increases; 5^*th*^ scenario, lockdown decreases; 6^*th*^ scenario, *SD* decreases; 7^*th*^ scenario, lockdown and *SD* increases; 8^*th*^ scenario, lockdown decreases and *SD* increases; 9^*th*^ scenario, *ρ* is the same as in Phase 1; 10^*th*^ scenario, *ρ* is the same as in Phase 1 while *SD* increases; 11^*th*^ scenario, *ρ* = 0.75 in all Phase 2; 12^*th*^ scenario, *ρ* = 0.75 while *SD* increases.

The results of applying the scenarios (1^*st*^–12^*th*^) to **Phase 2** is displayed in Tables 5 and 6 and presented graphically in Fig 8.

**Table 5:** Results of different scenarios for Phase 2, (1^*st*^- 6^*th*^) scenario.

Scenarios	*t*	*ρ*	*SD*	Peak(day)	Active Cases	Change Percentage	Notes
	*t*2	0.75	0.30	122	63,130	-	The fitting value
	*t*3	0.65	0.45				
	*t*4	0.60	0.55				
1^*st*^ scenario	*t*2	1	0.30	120	150,900	+139.03%	No lockdown enforcement
	*t*3	1	0.45				
	*t*4	1	0.55				
2^*nd*^ scenario	*t*2	1	0.33	121	128,600	+103.70%	Increase *SD* by 10%
	*t*3	1	0.495				
	*t*4	1	0.605				
	*t*2	1	0.39	121	79,380	+25.74%	Increase *SD* by 30%
	*t*3	1	0.585				
	*t*4	1	0.715				
	*t*2	1	0.42	122	57,630	−8.71%	Increase *SD* by 40%
	*t*3	1	0.63				
	*t*4	1	0.77				
3^*rd*^ scenario	*t*2	0.675	0.30	122	49,840	−21.05%	Increase lockdown by 10%
	*t*3	0.585	0.45				
	*t*4	0.54	0.55				
	*t*2	0.525	0.30	123	29,040	−53.99%	Increase lockdown by 30%
	*t*3	0.455	0.45				
	*t*4	0.42	0.55				
	*t*2	0.45	0.30	123	21,430	−66.05%	Increase lockdown by 40%
	*t*3	0.39	0.45				
	*t*4	0.36	0.55				
4^*th*^ scenario	*t*2	0.75	0.33	122	50,900	−19.37%	Increase *SD* by 10%
	*t*3	0.65	0.495				
	*t*4	0.60	0.605				
	*t*2	0.75	0.39	122,123	31,160	−50.64%	Increase *SD* by 30%
	*t*3	0.65	0.585				
	*t*4	0.60	0.715				
	*t*2	0.75	0.42	123	23,640	−62.55%	Increase *SD* by 40%
	*t*3	0.65	0.63				
	*t*4	0.60	0.77				
5^*th*^ scenario	*t*2	0.825	0.30	121	78,150	+23.79%	Decrease lockdown by 10%
	*t*3	0.715	0.45				
	*t*4	0.66	0.55				
	*t*2	0.975	0.30	121	111,700	+76.93%	Decrease lockdown by 30%
	*t*3	0.845	0.45				
	*t*4	0.78	0.55				
	*t*2	0.975	0.30	121	124,000	+96.42%	Decrease lockdown by 40%
	*t*3	0.91	0.45				
	*t*4	0.84	0.55				
6^*th*^ scenario	*t*2	0.75	0.27	121	76,750	+21.57%	Decrease *SD* by 10%
	*t*3	0.65	0.405				
	*t*4	0.60	0.495				
	*t*2	0.75	0.21	121	107,100	+69.64%	Decrease *SD* by 30%
	*t*3	0.65	0.315				
	*t*4	0.60	0.385				
	*t*2	0.75	0.18	121	122,500	+94.04%	Decrease *SD* by 40%
	*t*3	0.65	0.27				
	*t*4	0.60	0.33				

**Table 6:** Results of different scenarios for Phase 2, (7^*th*^–12^*th*^) scenario.

Scenarios	*t*	*ρ*	*SD*	Peak(day)	Active Cases	Change Percentage	Notes
	*t*2	0.75	0.30	122	63,130	-	The fitting value
	*t*3	0.65	0.45				
	*t*4	0.60	0.55				
7^*th*^ scenario	*t*2	0.675	0.33	122	40,350	−36.08%	Increase lockdown and *SD* by 10%
	*t*3	0.585	0.495				
	*t*4	0.54	0.605				
	*t*2	0.60	0.36	123	25,290	−59.93%	Increase lockdown and *SD* by 20%
	*t*3	0.52	0.54				
	*t*4	0.48	0.66				
	*t*2	0.525	0.39	123	15,820	−74.94%	Increase lockdown and *SD* by 30%
	*t*3	0.455	0.585				
	*t*4	0.42	0.715				
8^*th*^ scenario	*t*2	0.825	0.33	122	63,030	−0.15%	Decrease lockdown and increase *SD* by 10%
	*t*3	0.715	0.495				
	*t*4	0.66	0.605				
	*t*2	0.90	0.36	122	63,300	−4.48%	Decrease lockdown and increase *SD* by 20%
	*t*3	0.78	0.54				
	*t*4	0.72	0.66				
	*t*2	0.975	0.39	122	55,070	−12.76%	Decrease lockdown and increase *SD* by 30%
	*t*3	0.845	0.585				
	*t*4	0.78	0.715				
9^*th*^ scenario	*t*2	0.85	0.30	121	113,900	+80.42%	Lockdown as in Phase 1
	*t*3	0.85	0.45				
	*t*4	0.85	0.55				
10^*th*^ scenario	*t*2	0.85	0.33	121	92,830	+47.04%	Increase *SD* by 10%
	*t*3	0.85	0.495				
	*t*4	0.85	0.605				
	*t*2	0.85	0.36	122	72,850	+15.39%	Increase *SD* by 20%
	*t*3	0.85	0.54				
	*t*4	0.85	0.66				
	*t*2	0.85	0.39	122	55,160	−12.62%	Increase *SD* by 30%
	*t*3	0.85	0.585				
	*t*4	0.85	0.715				
	*t*2	0.85	0.42	122	40,220	−36.29%	Increase *SD* by 40%
	*t*3	0.85	0.63				
	*t*4	0.85	0.77				
	*t*2	0.85	0.45	123	28,210	−55.31%	Increase *SD* by 50%
	*t*3	0.85	0.675				
	*t*4	0.85	0.825				
11^*th*^ scenario	*t*2	0.75	0.30	121	87,550	+38.68%	Lockdown in all Phase 2 as in period *t*2
	*t*3	0.75	0.45				
	*t*4	0.75	0.55				
12^*th*^ scenario	*t*2	0.75	0.33	122	70,350	+11.43%	Increase *SD* by 10%
	*t*3	0.75	0.495				
	*t*4	0.75	0.605				
	*t*2	0.75	0.36	122	55,010	−12.86%	Increase *SD* by 20%
	*t*3	0.75	0.54				
	*t*4	0.75	0.66				
	*t*2	0.75	0.39	122	41,790	−33.80%	Increase *SD* by 30%
	*t*3	0.75	0.585				
	*t*4	0.75	0.715				
	*t*2	0.75	0.42	123	30,840	−51.14%	Increase *SD* by 40%
	*t*3	0.75	0.63				
	*t*4	0.75	0.77				
	*t*2	0.75	0.45	123	22,060	−65.05%	Increase *SD* by 50%
	*t*3	0.75	0.675				
	*t*4	0.75	0.825				

**Fig 9:**
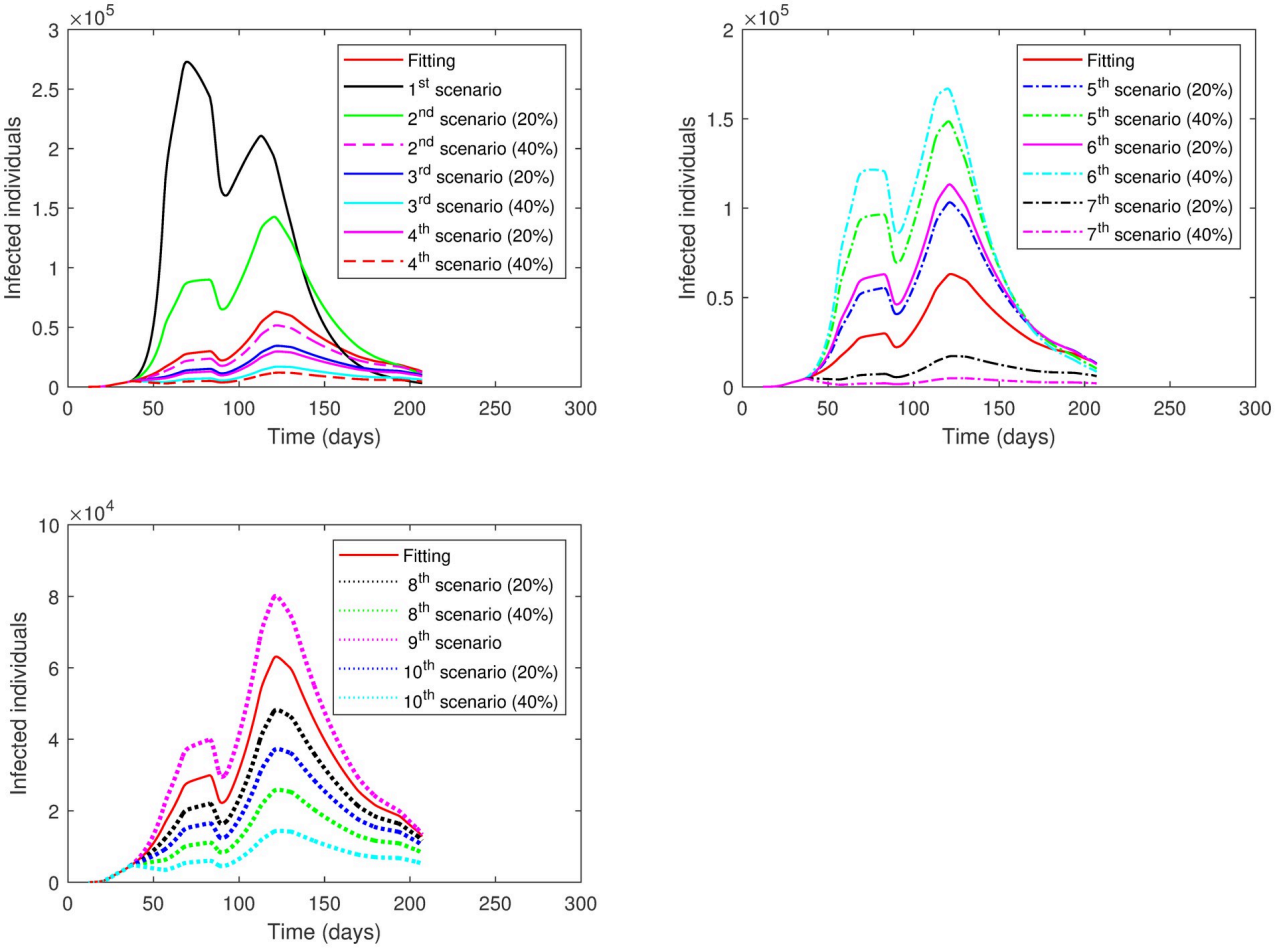
The numerical solution of model (1) for infected compartment vs. time with different scenarios of Phase 3 in the First Case: 1^*st*^ scenario, no lockdown is implemented; 2^*nd*^ scenario, no lockdown while *SD* increases; 3^*rd*^ scenario, lockdown increases; 4^*th*^ scenario, *SD* increases; 5^*th*^ scenario, lockdown decreases; 6^*th*^ scenario, *SD* decreases; 7^*th*^ scenario, lockdown and *SD* increases; 8^*th*^ scenario, lockdown decreases and *SD* increases; 9^*th*^ scenario, *ρ* is the same as in Phase 2; 10^*th*^ scenario, *ρ* is the same as in Phase 2 while *SD* increases.

The results of applying the scenarios (1^*st*^–10^*th*^) to **Phase 3** is exhibited in Table 7 and illustrated in Fig 9.

**Table 7:** Results of different scenarios for Phase 3.

Scenarios	*ρ*	*SD*	Peak(day)	Active Cases	Change Percentage	Notes
	0.55	0.55	122	63,130	-	The fitting value
1^*st*^ scenario	1	0.55	70	272,900	+332.28%	No lockdown enforcement
2^*nd*^ scenario	1	0.605	116	185,800	+194.13%	Increase *SD* by 10%
	1	0.66	120,121	142,700	+126.04%	Increase *SD* by 20%
	1	0.715	121	92,710	+46.85%	Increase *SD* by 30%
	1	0.77	122	51,600	−18.26%	Increase *SD* by 40%
	1	0.825	123	25,100	−60.24%	Increase *SD* by 50%
3^*rd*^ scenario	0.495	0.55	122	47,280	−25.10%	Increase lockdown by 10%
	0.44	0.55	122	34,460	−45.41%	Increase lockdown by 20%
	0.385	0.55	123	24,480	−61.22%	Increase lockdown by 30%
	0.33	0.55	123	16,940	−73.16%	Increase lockdown by 40%
4^*th*^ scenario	0.55	0.605	122	44,170	−30.03%	Increase *SD* by 10%
	0.55	0.66	122,123	29,690	−52.97%	Increase *SD* by 20%
	0.55	0.715	123	19,210	−69.57%	Increase *SD* by 30%
	0.55	0.77	123,124	11,930	−81.10%	Increase *SD* by 40%
5^*th*^ scenario	0.605	0.55	121	81,950	+29.81%	Decrease lockdown by 10%
	0.66	0.55	121	103,300	+63.63%	Decrease lockdown by 20%
	0.715	0.55	121	126,100	+99.74%	Decrease lockdown by 30%
	0.77	0.55	120	148,600	+135.38%	Decrease lockdown by 40%
6^*th*^ scenario	0.55	0.495	121	86,520	+37.05%	Decrease *SD* by 10%
	0.55	0.44	121	113,400	+79.62%	Decrease *SD* by 20%
	0.55	0.385	121	141,200	+123.66%	Decrease *SD* by 30%
	0.55	0.33	120	167,000	+164.53%	Decrease *SD* by 40%
7^*th*^ scenario	0.495	0.605	122	33,340	−47.18%	Increase lockdown and *SD* by 10%
	0.44	0.66	123	17,230	−72.70%	Increase lockdown and *SD* by 20%
	0.385	0.715	123	8,996	−85.75%	Increase lockdown and *SD* by 30%
	0.33	0.77	124	4,861	−92.30%	Increase lockdown and *SD* by 40%
8^*th*^ scenario	0.605	0.605	122	57,320	−9.20%	Decrease lockdown and increase *SD* by 10%
	0.66	0.66	122	48,240	−23.58%	Decrease lockdown and increase *SD* by 20%
	0.715	0.715	122	37,190	−41.08%	Decrease lockdown and increase *SD* by 30%
	0.77	0.77	123	25,860	−59.03%	Decrease lockdown and increase *SD* by 40%
9^*th*^ scenario	0.60	0.55	121	80,120	+26.91%	Lockdown as in Phase 2
10^*th*^ scenario	0.60	0.605	122	56,030	−11.24%	Increase *SD* by 10%
	0.60	0.66	122	37,300	−40.91%	Increase *SD* by 20%
	0.60	0.715	123	23,700	−62.45%	Increase *SD* by 30%
	0.60	0.77	123	14,370	−77.23%	Increase *SD* by 40%
	0.60	0.825	123,124	8,286	−86.87%	Increase *SD* by 50%

**Fig 10:**
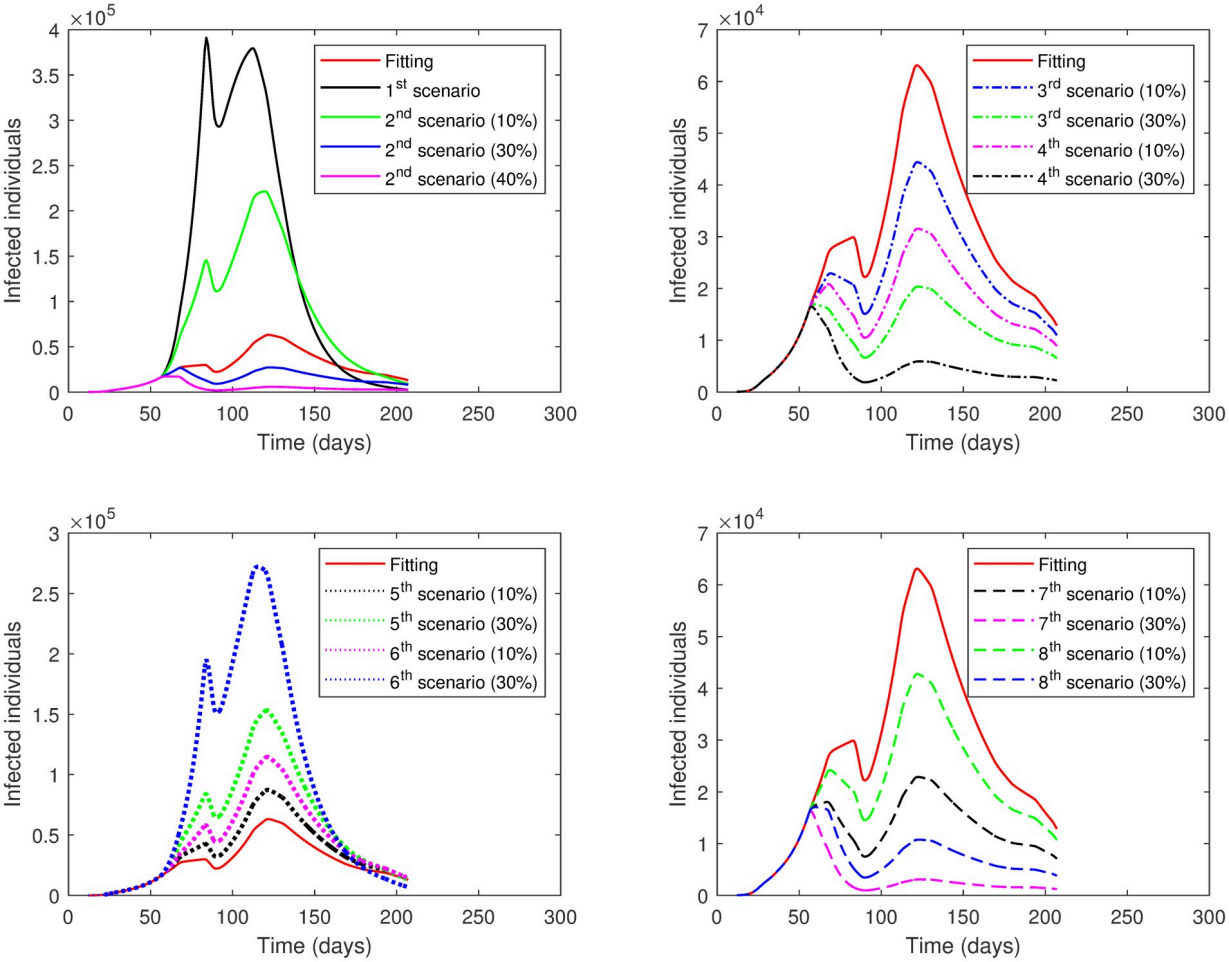
The numerical solution of model (1) for infected compartment vs. time with different scenarios of Phase 4 in the First Case: 1^*st*^ scenario, no lockdown is implemented; 2^*nd*^ scenario, no lockdown while *SD* increases; 3^*rd*^ scenario, lockdown increases; 4^*th*^ scenario, *SD* increases; 5^*th*^ scenario, lockdown decreases; 6^*th*^ scenario, *SD* decreases; 7^*th*^ scenario, lockdown and *SD* increases; 8^*th*^ scenario, lockdown decreases and *SD* increases.

The results of applying scenarios (1^*st*^–8^*th*^) to **Phase 4** are presented in Table 8 and shown graphically in Fig 10.

**Table 8:** Results of different scenarios for Phase 4.

Scenarios	*t*	*ρ*	*SD*	Peak(day)	Active Cases	Change Percentage	Notes
	*t*6	0.55	0.60	122	63,130	-	The fitting value
	*t*7	0.55	0.69				
1^*st*^ scenario	*t*6	1	0.60	84	391,100	+519.51%	No lockdown enforcement
	*t*7	1	0.69				
2^*nd*^ scenario	*t*6	1	0.66	120	221,700	+251.18%	Increase *SD* by 10%
	*t*7	1	0.759				
	*t*6	1	0.72	121	92,800	+46.99%	Increase *SD* by 20%
	*t*7	1	0.828				
	*t*6	1	0.78	122	27,150	−56.99%	Increase *SD* by 30%
	*t*7	1	0.897				
	*t*6	1	0.84	60,61	17,150	−72.83%	Increase *SD* by 40%
	*t*7	1	0.966				
3^*rd*^ scenario	*t*6	0.495	0.60	122	44,410	−29.65%	Increase lockdown by 10%
	*t*7	0.495	0.69				
	*t*6	0.44	0.60	122	30,450	−51.76%	Increase lockdown by 20%
	*t*7	0.44	0.69				
	*t*6	0.385	0.60	123	20,370	−67.73%	Increase lockdown by 30%
	*t*7	0.385	0.69				
4^*th*^ scenario	*t*6	0.55	0.66	122	31,540	−50.03%	Increase *SD* by 10%
	*t*7	0.55	0.759				
	*t*6	0.55	0.72	59	16,960	−73.13%	Increase *SD* by 20%
	*t*7	0.55	0.828				
	*t*6	0.55	0.78	57	16,500	−73.86%	Increase *SD* by 30%
	*t*7	0.55	0.897				
5^*th*^ scenario	*t*6	0.605	0.60	121	87,360	+38.38%	Decrease lockdown by 10%
	*t*7	0.605	0.69				
	*t*6	0.66	0.60	121	117,600	+86.28%	Decrease lockdown by 20%
	*t*7	0.66	0.69				
	*t*6	0.715	0.60	121	153,400	+142.99%	Decrease lockdown by 30%
	*t*7	0.715	0.69				
6^*th*^ scenario	*t*6	0.55	0.54	121	114,800	+81.84%	Decrease *SD* by 10%
	*t*7	0.55	0.621				
	*t*6	0.55	0.48	120	186,900	+196.05%	Decrease *SD* by 20%
	*t*7	0.55	0.552				
	*t*6	0.55	0.42	115	272,100	+331.01%	Decrease *SD* by 30%
	*t*7	0.55	0.483				
7^*th*^ scenario	*t*6	0.495	0.66	122,123	22,870	−63.77%	Increase lockdown and *SD* by 10%
	*t*7	0.495	0.759				
	*t*6	0.44	0.72	58	16,510	−73.84%	Increase lockdown and *SD* by 20%
	*t*7	0.44	0.828				
	*t*6	0.385	0.78	57	16,360	−74.08%	Increase lockdown and *SD* by 30%
	*t*7	0.385	0.897				
8^*th*^ scenario	*t*6	0.605	0.66	122	42,790	−32.21%	Decrease lockdown and increase *SD* by 10%
	*t*7	0.605	0.759				
	*t*6	0.66	0.72	122,123	24,090	−61.84%	Decrease lockdown and increase *SD* by 20%
	*t*7	0.66	0.828				
	*t*6	0.715	0.78	60	17,050	−72.99%	Decrease lockdown and increase *SD* by 30%
	*t*7	0.715	0.897				

At Phase 5, full lockdown (24-hours) was imposed in all cities of Saudi Arabia. Therefore, we added the following scenario:

13^*th*^ **scenario**: This scenario analyzes the effect of decreasing lockdown and social distancing (*SD*) simultaneously.

**Fig 11:**
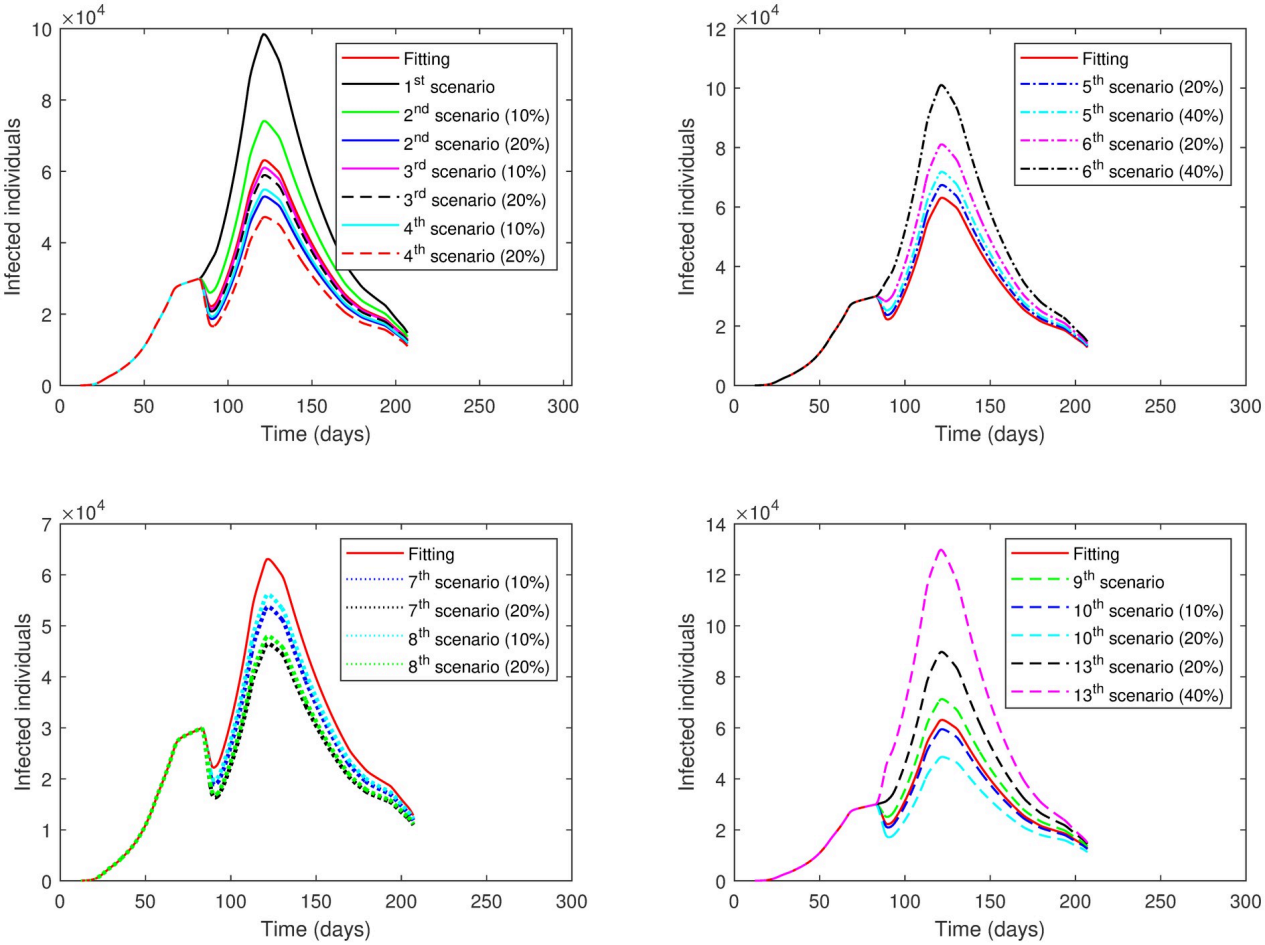
The numerical solution of model (1) for infected compartment vs. time with different scenarios of Phase 5 in the First Case: 1^*st*^ scenario, no lockdown is implemented; 2^*nd*^ scenario, no lockdown while *SD* increases; 3^*rd*^ scenario, lockdown increases; 4^*th*^ scenario, *SD* increases; 5^*th*^ scenario, lockdown decreases; 6^*th*^ scenario, *SD* decreases; 7^*th*^ scenario, lockdown and *SD* increases; 8^*th*^ scenario, lockdown decreases and *SD* increases; 9^*th*^ scenario, *ρ* is the same as in Phase 4; 10^*th*^ scenario, *ρ* is the same as in Phase 4 while *SD* increases; 13^*th*^ scenario, lockdown and *SD* decrease.

The results of applying the scenarios (1^*st*^–10^*th*^, 13^*th*^) to **Phase 5** is displayed in Table 9 and illustrated in Fig 11.

**Table 9:** Results of different scenarios for Phase 5.

Scenarios	*ρ*	*SD*	Peak(day)	Active Cases	Change Percentage	Notes
	0.40	0.80	122	63,130	-	The fitting value
1^*st*^ scenario	1	0.80	121	98,420	+55.90%	No lockdown enforcement
2^*nd*^ scenario	1	0.88	122	74,090	+17.36%	Increase *SD* by 10%
	1	0.96	122	52,950	−16.12%	Increase *SD* by 20%
3^*rd*^ scenario	0.36	0.80	122	61,030	−3.32%	Increase lockdown by 10%
	0.32	0.80	122	58,960	−6.60%	Increase lockdown by 20%
	0.28	0.80	122	56,920	−9.83%	Increase lockdown by 30%
	0.24	0.80	122	54,920	−13.004%	Increase lockdown by 40%
4^*th*^ scenario	0.40	0.88	122	54,920	−13.004%	Increase *SD* by 10%
	0.40	0.96	122	47,220	−25.20%	Increase *SD* by 20%
5^*th*^ scenario	0.44	0.80	122	65,260	+3.37%	Decrease lockdown by 10%
	0.48	0.80	122	67,420	+6.79%	Decrease lockdown by 20%
	0.52	0.80	122	69,610	+10.26%	Decrease lockdown by 30%
	0.56	0.80	122	71,830	+13.78%	Decrease lockdown by 40%
6^*th*^ scenario	0.40	0.72	122	71,830	+13.78%	Decrease *SD* by 10%
	0.40	0.64	122	81,040	+28.37%	Decrease *SD* by 20%
	0.40	0.56	121	90,780	+43.79%	Decrease *SD* by 30%
	0.40	0.48	121	101,000	+59.98%	Decrease *SD* by 40%
7^*th*^ scenario	0.36	0.88	122	53,730	−14.88%	Increase lockdown and *SD* by 10%
	0.32	0.96	122	46,480	−26.37%	Increase lockdown and *SD* by 20%
	0.28	0.96	122	46,110	−26.96%	Increase lockdown and *SD* by 30%
	0.24	0.96	122	45,740	−27.54%	Increase lockdown and *SD* by 40%
8^*th*^ scenario	0.44	0.88	122	56,120	−11.10%	Decrease lockdown and increase *SD* by 10%
	0.48	0.96	122	47,970	−24.01%	Decrease lockdown and increase *SD* by 20%
	0.52	0.96	122	48,340	−23.42%	Decrease lockdown and increase *SD* by 30%
	0.56	0.96	122	48,720	−22.82%	Decrease lockdown and increase *SD* by 40%
9^*th*^ scenario	0.55	0.80	122	71,270	+12.89%	Lockdown as in Phase 4
10^*th*^ scenario	0.55	0.88	122	59,470	−5.79%	Increase *SD* by 10%
	0.55	0.96	122	48,620	−22.98%	Increase *SD* by 20%
13^*th*^ scenario	0.44	0.72	122	75,000	+18.80%	Decrease lockdown and *SD* by 10%
	0.48	0.64	121	89,780	+42.21%	Decrease lockdown and *SD* by 20%
	0.52	0.56	121	107,900	+70.91%	Decrease lockdown and *SD* by 30%
	0.56	0.48	121	129,900	+105.76%	Decrease lockdown and *SD* by 40%

**Fig 12:**
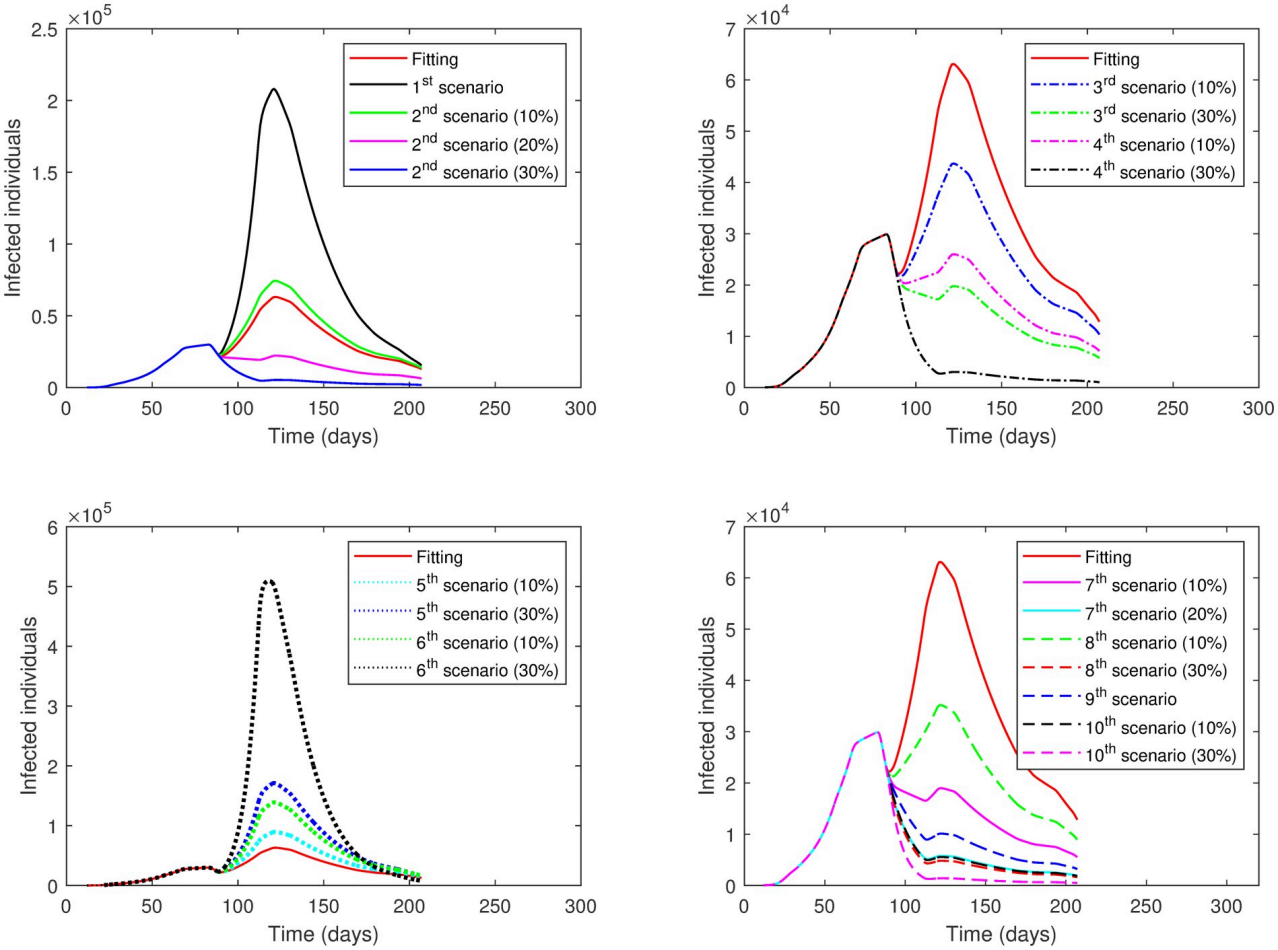
The numerical solution of model (1) for infected compartment vs. time with different scenarios of Phase 6 in the First Case: 1^*st*^ scenario, no lockdown is implemented; 2^*nd*^ scenario, no lockdown while *SD* increases; 3^*rd*^ scenario, lockdown increases; 4^*th*^ scenario, *SD* increases; 5^*th*^ scenario, lockdown decreases; 6^*th*^ scenario, *SD* decreases; 7^*th*^ scenario, lockdown and *SD* increases; 8^*th*^ scenario, lockdown decreases and *SD* increases; 9^*th*^ scenario, *ρ* is the same as in Phase 5; 10^*th*^ scenario, *ρ* is the same as in Phase 5 while *SD* increases.

The results of applying the scenarios (1^*st*^–10^*th*^) to **Phase 6** are exhibited in Table 10 and presented graphically in Fig 12.

**Table 10:** Results of different scenarios for Phase 6.

Scenarios	*t*	*ρ*	*SD*	Peak(day)	Active Cases	Change Percentage	Notes
	*t*9	0.65	0.70	122	63,130	-	The fitting value
	*t*10	0.75	0.70				
1^*st*^ scenario	*t*9	1	0.70	121	208,100	+229.63%	No lockdown enforcement
	*t*10	1	0.70				
2^*nd*^ scenario	*t*9	1	0.77	122	74,370	+17.80%	Increase *SD* by 10%
	*t*10	1	0.77				
	*t*9	1	0.84	83	29,970	−52.52%	Increase *SD* by 20%
	*t*10	1	0.84				
	*t*9	1	0.91	83	29,970	−52.52%	Increase *SD* by 30%
	*t*10	1	0.91				
3^*rd*^ scenario	*t*9	0.585	0.70	122	43,710	−30.76%	Increase lockdown by 10%
	*t*10	0.675	0.70				
	*t*9	0.455	0.70	83	29,970	−52.52%	Increase lockdown by 30%
	*t*10	0.525	0.70				
4^*th*^ scenario	*t*9	0.65	0.77	83	29,970	−52.52%	Increase *SD* by 10%
	*t*10	0.75	0.77				
	*t*9	0.65	0.91	83	29,970	−52.52%	Increase *SD* by 30%
	*t*10	0.75	0.91				
5^*th*^ scenario	*t*9	0.715	0.70	121,122	89,540	+41.83%	Decrease lockdown by 10%
	*t*10	0.825	0.70				
	*t*9	0.78	0.70	121	125,000	+98.004%	Decrease lockdown by 30%
	*t*10	0.90	0.70				
	*t*9	0.845	0.70	121	171,400	+171.50%	Decrease lockdown by 30%
	*t*10	0.975	0.70				
6^*th*^ scenario	*t*9	0.65	0.63	121	139,100	+120.33%	Decrease *SD* by 10%
	*t*10	0.75	0.63				
	*t*9	0.65	0.56	121	278,700	+341.46%	Decrease *SD* by 20%
	*t*10	0.75	0.56				
	*t*9	0.65	0.49	118	509,200	+706.58%	Decrease *SD* by 30%
	*t*10	0.75	0.49				
7^*th*^ scenario	*t*9	0.585	0.77	83	29,970	−52.52%	Increase lockdown and *SD* by 10%
	*t*10	0.675	0.77				
	*t*9	0.52	0.84	83	29,970	−52.52%	Increase lockdown and *SD* by 20%
	*t*10	0.60	0.84				
8^*th*^ scenario	*t*9	0.715	0.77	122	35,200	−44.24%	Decrease lockdown and increase *SD* by 10%
	*t*10	0.825	0.77				
	*t*9	0.845	0.91	83	29,970	−52.52%	Decrease lockdown and increase *SD* by 30%
	*t*10	0.975	0.91				
9^*th*^ scenario	*t*9	0.40	0.70	83	29,970	−52.52%	Lockdown as in Phase 5
	*t*10	0.40	0.70				
10^*th*^ scenario	*t*9	0.40	0.77	83	29,970	−52.52%	Increase *SD* by 10%
	*t*10	0.40	0.77				
	*t*9	0.40	0.91	83	29,970	−52.52%	Increase *SD* by 30%
	*t*10	0.40	0.91				

The analysis of **First Case** can be summarized as follows. We applied different scenarios for lockdown (*ρ*) and social distancing (*SD*) for each phase separately. At the selected phase, the values of *ρ* and *SD* were varied, but they are kept the same as in the fitting values in other phases. The aim of the 1^*st*^ scenario was to examine the effect of no lockdown in each phase (Phase 1—Phase 6). The results in this scenario yielded a rise in the number of active cases compared to the actual data with an increasing percentage ranging from (50.32%—519.51%). This indicates the significance of lockdown enforcement.

In the 2^*nd*^ scenario, the focus was to determine whether it is possible to rely on social distancing only in the absence of lockdown. When implementing this scenario in each phase, we varied the social distancing value. We concluded that to reduce the number of active cases in the actual data, we must increase the values of social distancing. We found that in Phase 1, we needed to increase social distancing to 90% or more. Whereas in Phase 2 and Phase 3, the social distancing must increase to 40% or more, and in Phase 4, to 30% or more. However, the increased percentage of social distancing is less in Phase 5 and Phase 6, which is 20% or more.

In the 3^*rd*^ and 4^*th*^ scenarios, the plan was to discover the most effective control measures. Is it the lockdown applications or the social distancing implementations? We increased the lockdown enforcement in the 3^*rd*^ scenario and kept the values of the social distancing as the fitting. Conversely, in the 4^*th*^ scenario, we increased the social distancing values and kept lockdown values as in the fitting. In both scenarios, the active cases decreased when the control measures increased by 10% or more, confirming the effectiveness of both measures.

Following the same plan as in the previous scenarios, but with reduced control procedures, we decreased the lockdown enforcement in the 5^*th*^ scenario while keeping the social distancing values the same as in the fitting. On the contrary, in the 6^*th*^ scenario, we kept lockdown values as in the fitting and decreased the social distancing values. As a result, the active cases increased in each phase when the control measures decreased by 10% or more. Moreover, when comparing the number of active cases in each phase separately (see Tables 4–10), we found that Phase 1 and Phase 2 are affected more by the changes in the lockdown application than the changes in social distancing. On the other hand, Phases 3 through 6 are most affected by the changes in social distancing implementation.

In the 7^*th*^ scenario, we increased the lockdown enforcement and social distancing simultaneously. As expected, the active cases decreased in all phases when applying this scenario by 10% or more. This means that both measures complement each other.

In the 8^*th*^ scenario, we checked the reliability of the practice of social distancing again. In this scenario, we reduced the lockdown application and increased the execution of social distancing by the same percentage. We found that to decrease the number of active cases in Phase 1, the percentage must be 60% or more, and in Phase 2, the percentage must be 20% or more. As for the rest phases, a percentage of 10% or more can decrease the number of active cases compared to the actual data.

The scenarios above were applied to all phases separately, while the following were applied in Phases: 2, 3, 5 and 6.

In the 9^*th*^ and 10^*th*^ scenarios, we explored the effect of the continued lockdown level from the previous phase. In the 9^*th*^ scenario, we kept the social distancing values as in the fitting. We found that the number of active cases increased when applying this scenario to Phases: 2, 3, and 5 since the level of lockdown was lower in the previous phases. However, a decreasing number of active cases resulted in Phase 6 since the level of lockdown in Phase 5 was higher than in Phase 6. In the 10^*th*^ scenario, we increased the social distancing values. As a result, active cases decreased when increasing social distancing by 30% or more in Phase 2 and by 10% or more in Phases: 3, 5, and 6. This demonstrates that the changes in lockdown measures during the phases are significant.

The 11^*th*^ and 12^*th*^ scenarios were applied only in Phase 2 since this phase is divided into three time periods. Each period had a different decision. The 11^*th*^ scenario examined the effect of applying one decision for the entire phase. The decision was to implement partial lockdown for only 11-hours while keeping the values of the social distancing as in the fitting. This increased the number of active cases by 38.68% compared to the actual data. The same decision was applied in the 12^*th*^ scenario but with increasing values of the social distancing. We found that the number of active cases declined when increasing the social distancing by 20% or more.

Finally, the 13^*th*^ scenario was applied on Phase 5. At this phase, the decision was complete lockdown for 24-hours in all cities. The scenario investigated the relaxation in control measures during this phase. Both values of lockdown and social distancing were decreased simultaneously. The result was an increase in the number of active cases even when the measures were decreased by just 10%. This indicates the importance of the complete lockdown decision in this critical phase.

**Second Case**: Different scenarios are presented for all phases (Phase 1—Phase 7) at once. In each scenario, we take a fixed level of lockdown to be applied in all phases. Four scenarios are investigated as follows:

1^*st*^ **scenario**: This scenario analyzes the effect of no lockdown enforcement (*ρ* = 1) in all phases.
2^*nd*^ **scenario**: This scenario analyzes the effect of applying the level of lockdown from Phase 1 to all phases, that is, *ρ* = 0.85.
3^*rd*^ **scenario**: This scenario analyzes the effect of applying the level of lockdown from Phase 2 to all phases, that is, *ρ* = 0.75.
4^*th*^ **scenario**: This scenario analyzes the effect of applying the level of lockdown from Phase 4 to all phases, that is, *ρ* = 0.55.

Moreover, in each scenario, we apply seven events (I—VII) regarding varying social distancing values:

**Event I**: The value of social distancing (*SD*) is the same as the fitting value for each phase.
**Event I**: The value of social distancing (*SD*) increases by 10% in each phase.
**Event III**: The value of social distancing (*SD*) increases by 20% in each phase.
**Event IV**: The value of social distancing (*SD*) increases by 30% in each phase.
**Event V**: The value of social distancing (*SD*) increases by 40% in each phase.
**Event VI**: The value of social distancing (*SD*) increases by 50% in each phase.
**Event VII**: The value of social distancing (*SD*) increases by 60% in each phase.

**Fig 13:**
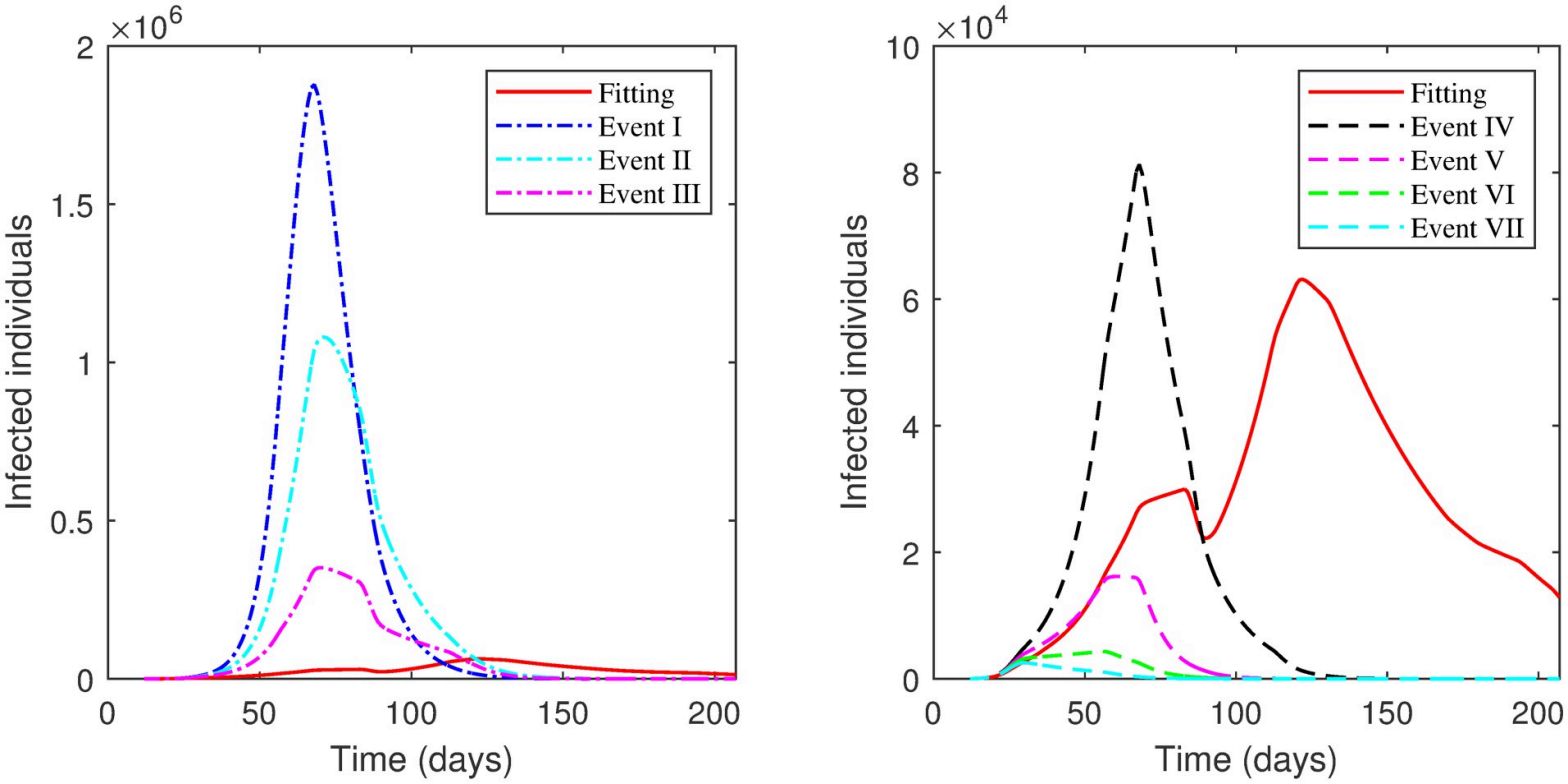
Numerical solution of model (1) for infected compartment vs. time for 1^*st*^ scenario of Second Case.

**Fig 14:**
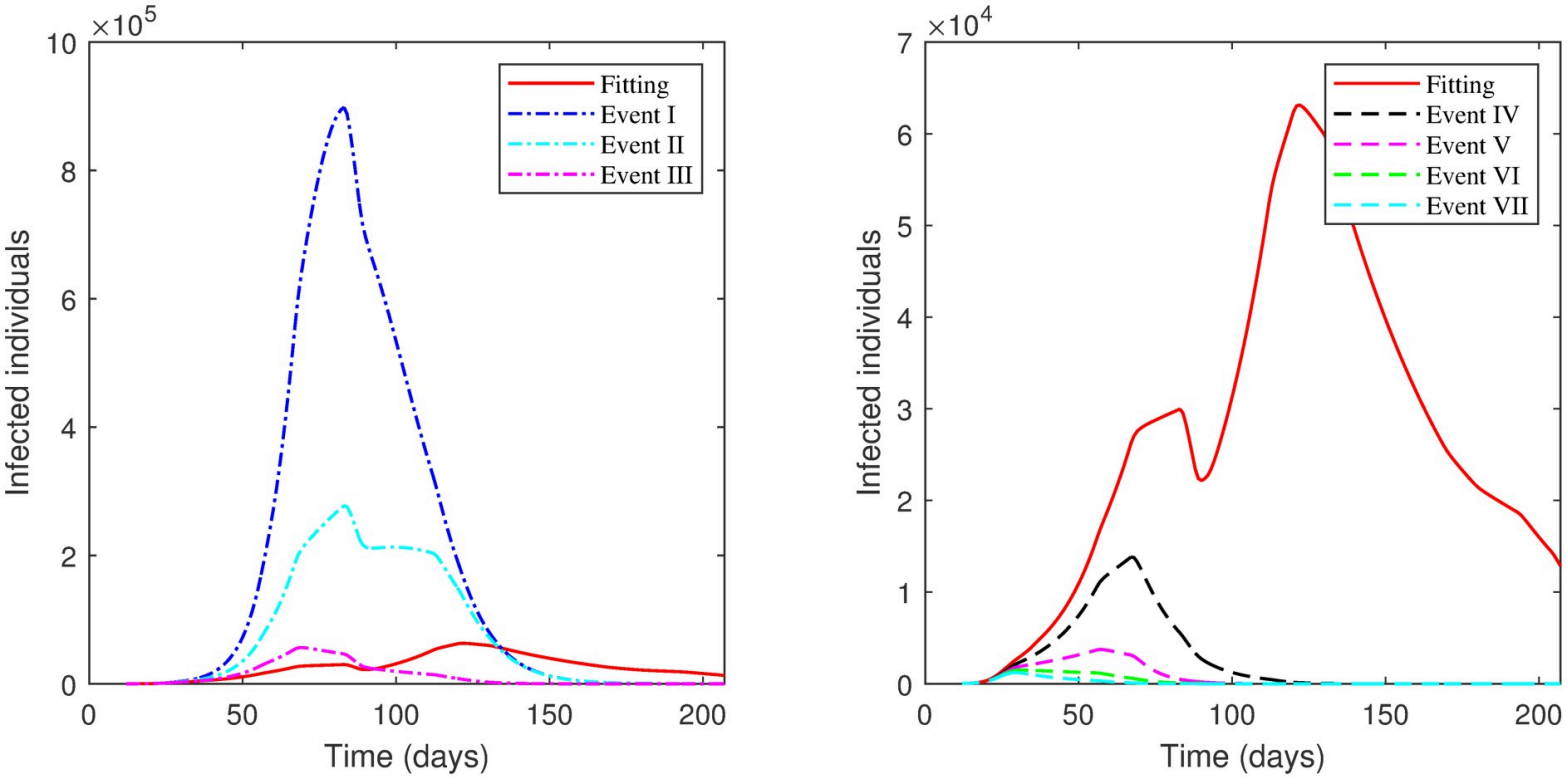
Numerical solution of model (1) for infected compartment vs. time for 2^*nd*^ scenario of Second Case.

**Fig 15:**
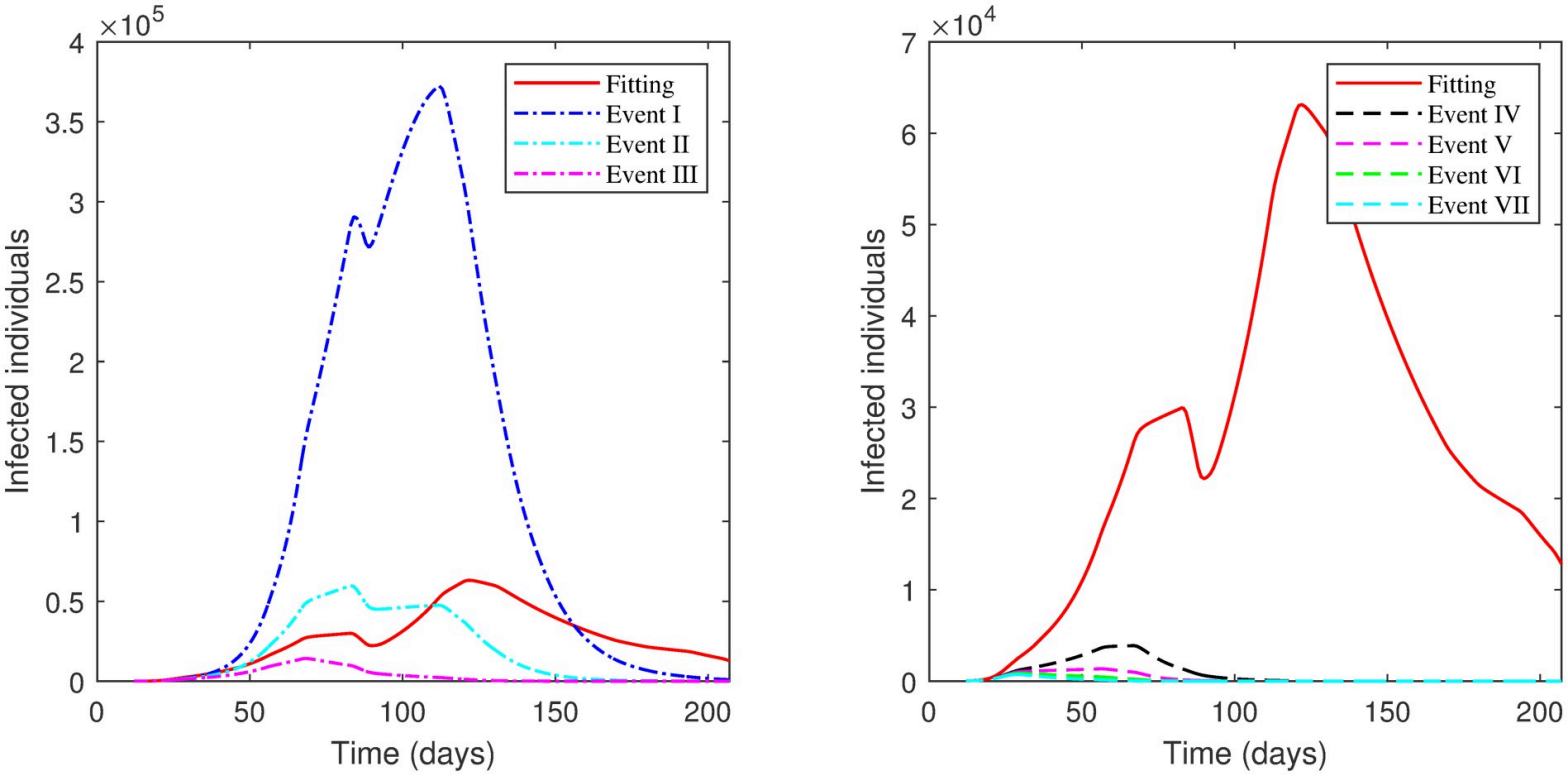
Numerical solution of model (1) for infected compartment vs. time for 3^*rd*^ scenario of Second Case.

**Fig 16:**
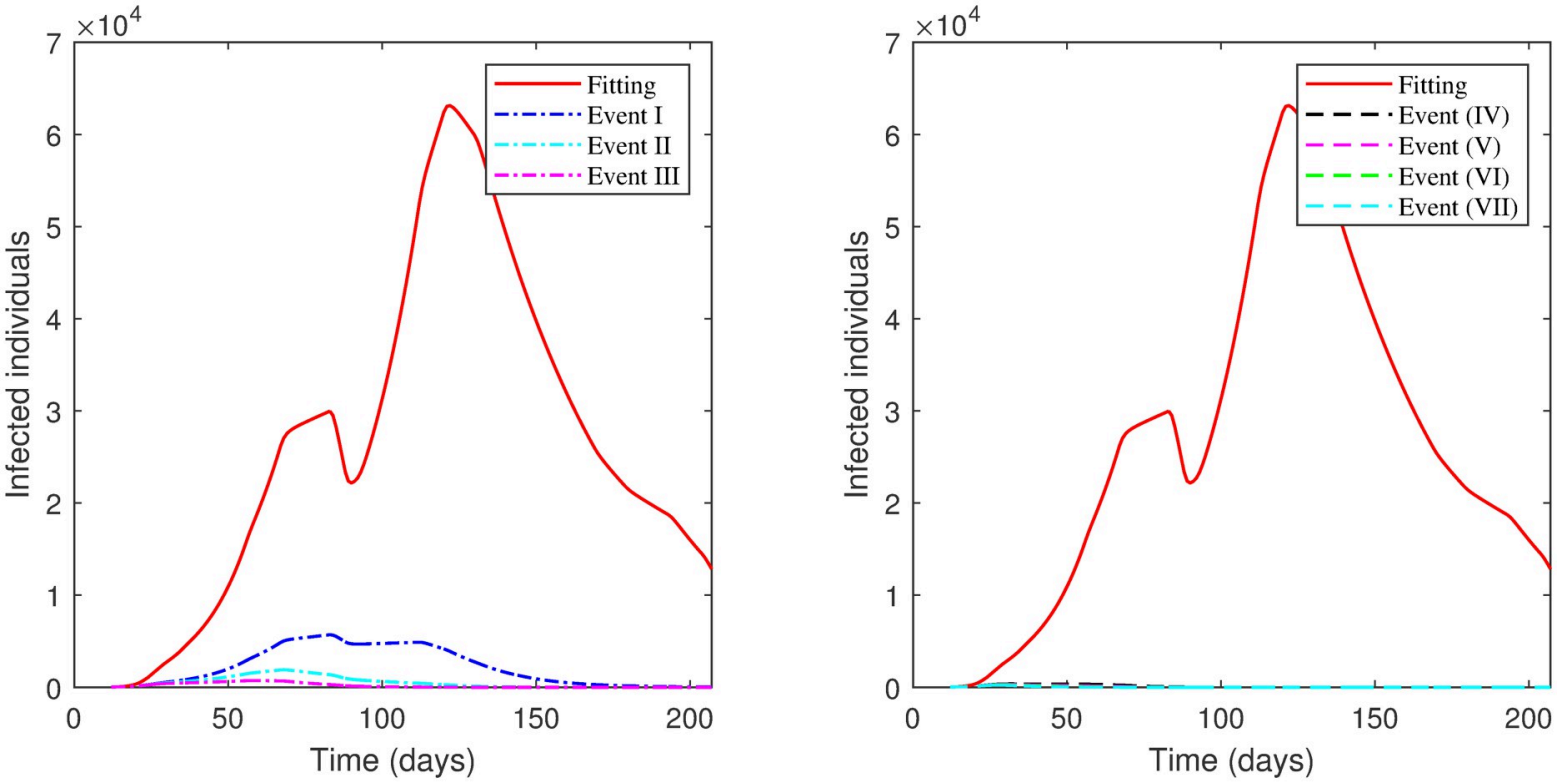
Numerical solution of model (1) for infected compartment vs. time for 4^*th*^ scenario of Second Case.

The results of each scenario (1^*st*^ scenario—4^*th*^ scenario) are displayed in Tables 11–14, and graphically in Figs 13–16.

**Table 11:** Results of different scenarios Second Case, 1^*st*^ scenario.

Events	*ρ*	*SD*	Peak(day)	Active cases	Change Percentage
I	1	*SD* in fitting	68	1,878,000	+2874.81%
II	1	Increase *SD* by 10%	71	1,080,000	+1610.75%
III	1	Increase *SD* by 20%	70	351,600	+456.94%
IV	1	Increase *SD* by 30%	68	81,270	+28.73%
V	1	Increase *SD* by 40%	60,61	16,170	−74.38
VI	1	Increase *SD* by 50%	56	4,326	−93.14
VII	1	Increase *SD* by 60%	30	2,542	−95.97

**Table 12:** Results of different scenarios Second Case, 2^*nd*^ scenario.

Events	*ρ*	*SD*	Peak(day)	Active cases	Change Percentage
I	0.85	*SD* in fitting	83	898,400	+1323.09%
II	0.85	Increase *SD* by 10%	83	277,600	+339.72%
III	0.85	Increase *SD* by 20%	69	56,450	−10.58%
IV	0.85	Increase *SD* by 30%	67	13,830	−78.09%
V	0.85	Increase *SD* by 40%	57	3,750	−94.05%
VI	0.85	Increase *SD* by 50%	31	1,509	−97.60%
VII	0.85	Increase *SD* by 60%	29	1,234	−98.04%

**Table 13:** Results of different scenarios Second Case, 3^*rd*^ scenario.

Events	*ρ*	*SD*	Peak(day)	Active cases	Change Percentage
I	0.75	*SD* in fitting	112	372,400	+489.89%
II	0.75	Increase *SD* by 10%	83	59,810	−5.25%
III	0.75	Increase *SD* by 20%	68	14,220	−77.47%
IV	0.75	Increase *SD* by 30%	67	3,897	−93.82%
V	0.75	Increase *SD* by 40%	57	1,352	−97.85%
VI	0.75	Increase *SD* by 50%	30	878	−98.60%
VII	0.75	Increase *SD* by 60%	29	743	−98.82%

**Table 14:** Results of different scenarios Second Case, 4^*th*^ scenario.

Events	*ρ*	*SD*	Peak(day)	Active cases	Change Percentage
I	0.55	*SD* in fitting	83	5,709	−90.95%
II	0.55	Increase *SD* by 10%	68	1,906	−96.98%
III	0.55	Increase *SD* by 20%	58	732	−98.84%
IV	0.55	Increase *SD* by 30%	32	368	−99.41%
V	0.55	Increase *SD* by 40%	29	317	−99.49%
VI	0.55	Increase *SD* by 50%	29	279	−99.55%
VII	0.55	Increase *SD* by 60%	28	249	−99.60%

The examination of the scenarios in the **Second Case** reveals the following. In the 1^*st*^ scenario where the lockdown was absent, the active cases increased to a very high peak. We had to increase the social distancing value by 40% across all phases to reach a lower number of active cases below the number in the actual data. This indicates the importance of lockdown enforcement.

In the 2^*nd*^ scenario where the lockdown level was the same as in Phase 1, the number of active cases decreased when we increased the value of social distancing by 20% or more across all phases. The major decisions that the government of Saudi Arabia imposed in this scenario were to close its borders, schools, universities, and workplaces. This means that if only these procedures were implemented, social distancing must be achieved by 20% or more at all phases to acquire a lower number of active cases.

The 3^*rd*^ scenario shows that only up to a 10% increase in social distancing values across all phases was needed to reduce the number of active cases if a partial lockdown of 11-hours was imposed as in Phase 2 alongside implemented measures from Phase 1. On the other hand, there was no need to increase social distancing values across phases to decrease the number of active cases if a partial lockdown of 16-hours was enforced as in Phase 4 adjacent to executed measures from Phase 1 as shown in the 4^*th*^ scenario.

In the actual data, the peak of active cases occurred on day 122. In the Second Case scenarios, the peak occurred before this day regardless of the peak value; it may be higher or lower than the value in the actual data. However, in the First Case scenarios, the peak day is nearly the same as the actual data.

We conclude from the Second Case scenarios that increasing lockdown enforcement reduces the number of active cases. However, if lockdown is implemented at a certain level, social distancing must be increased to decrease the number of active cases.

## 5 Conclusion

A mathematical model was formulated to study the impact of lockdown and social distancing on the spread of COVID-19 in Saudi Arabia. The qualitative analysis of the model showed that the model is well-posed and has two equilibrium points: COVID-19 free equilibrium *P*_0_ and COVID-19 endemic equilibrium *P*_1_. The existence and stability of the equilibrium points depend on the threshold quantity $R_{c}$, the control reproduction number. The COVID-19 free equilibrium always exists, and it is locally and globally asymptotically stable if $R_{c}<1$. Whereas if $R_{c}>1$, the COVID-19 endemic equilibrium exists, and it is locally and globally asymptotically stable.

The model was fitted numerically to the available data on the COVID-19 dashboard of the Saudi Ministry of Health from March 12, 2020, till September 23, 2020. The fitting was performed to validate the model and estimate some of its parameters. The other parameters were either estimated intuitively or obtained from the literature. Moreover, the numerical experiments illustrated the consistency between the numerical solution and the qualitative analysis of the model. In addition, the sensitivity analysis for $R_{c}$ showed that the lockdown (*ρ*) and social distancing (*SD*) are of the most influential parameters in $R_{c}$. Finally, we investigated numerically different scenarios for lockdown and social distancing measures applied in Saudi Arabia to contain COVID-19.

We concluded that the two measures, namely, lockdown and social distancing, are significant, effective, and complement each other. Also, the changes in lockdown measures during the phases are significant. Moreover, we found that Phase 1 and Phase 2 are affected more by the changes in the lockdown application than the changes in social distancing. Conversely, Phases 3 through 6 are most affected by the changes in social distancing implementation. As a result, the implementation of lockdown is more critical at the beginning of the spread of the disease. Later, when community members become aware of the disease, lockdown may be eased with expanded social distancing measures such as wearing face masks, using sterilizers, and other preventive measures.
